# Integration of the Unfolded Protein and Oxidative Stress Responses through SKN-1/Nrf

**DOI:** 10.1371/journal.pgen.1003701

**Published:** 2013-09-12

**Authors:** Kira M. Glover-Cutter, Stephanie Lin, T. Keith Blackwell

**Affiliations:** 1 Joslin Diabetes Center, Harvard Stem Cell Institute, and Harvard Medical School Department of Genetics, Boston, Massachusetts, United States of America; The University of Texas Health Science Center at Houston, United States of America

## Abstract

The Unfolded Protein Response (UPR) maintains homeostasis in the endoplasmic reticulum (ER) and defends against ER stress, an underlying factor in various human diseases. During the UPR, numerous genes are activated that sustain and protect the ER. These responses are known to involve the canonical UPR transcription factors XBP1, ATF4, and ATF6. Here, we show in *C. elegans* that the conserved stress defense factor SKN-1/Nrf plays a central and essential role in the transcriptional UPR. While SKN-1/Nrf has a well-established function in protection against oxidative and xenobiotic stress, we find that it also mobilizes an overlapping but distinct response to ER stress. SKN-1/Nrf is regulated by the UPR, directly controls UPR signaling and transcription factor genes, binds to common downstream targets with XBP-1 and ATF-6, and is present at the ER. SKN-1/Nrf is also essential for resistance to ER stress, including reductive stress. Remarkably, SKN-1/Nrf-mediated responses to oxidative stress depend upon signaling from the ER. We conclude that SKN-1/Nrf plays a critical role in the UPR, but orchestrates a distinct oxidative stress response that is licensed by ER signaling. Regulatory integration through SKN-1/Nrf may coordinate ER and cytoplasmic homeostasis.

## Author Summary

Proteins that are placed in membranes or secreted are produced in a cellular structure called the endoplasmic reticulum (ER). An accumulation of misfolded proteins in the ER contributes to many disease states, including diabetes and neurodegeneration. The ER protects against a toxic buildup of misfolded proteins by activating the unfolded protein response (UPR), which maintains ER homeostasis by slowing protein synthesis and enhancing ER functions such as protein folding and degradation. Many of these processes are controlled by three canonical ER/UPR gene regulatory factors. Here we identify the gene regulator SKN-1/Nrf as also playing a critical role in the UPR. SKN-1/Nrf is well known for its functions in oxidative stress defense and longevity. We now report that SKN-1/Nrf mobilizes an ER stress gene network that is distinct from its oxidative stress response, and includes regulation of other central UPR factors. Surprisingly, we also find that ER- and UPR-associated mechanisms are needed to “license” SKN-1/Nrf to defend against oxidative stresses. Our findings show that UPR and oxidative stress defense mechanisms are integrated through SKN-1/Nrf, and suggest that this integration may help maintain a healthy balance between ER and cytoplasmic functions, and stress defenses.

## Introduction

The endoplasmic reticulum (ER) is responsible for multiple functions in protein synthesis and processing, lipid metabolism, xeno/endobiotic detoxification, and Ca^2+^ storage (reviewed in [1], [2]). The ER forms a continuous structure with the nuclear envelope and maintains extensive contact with mitochondria [3], [4]. Consequently, the ER is well positioned to sense and respond to changes in the cellular environment.

All secretory and membrane-bound proteins are synthesized in the rough ER, a process that is highly regulated so that only properly folded and modified proteins are released to the Golgi [1], [2], [5], [6]. Maturation and folding of these proteins involves glycosylation and formation of appropriate Cys-Cys crosslinks. When its protein folding capacity is exceeded (ER stress), the ER protects itself through the Unfolded Protein Response (UPR) (Figure S1A) [2], [5], [6]. This signaling and transcription program decreases protein translation, expands ER size and folding capacity, and directs misfolded proteins to be degraded in the cytosol. The UPR functions continuously to maintain ER homeostasis, but is amplified and diversified under ER stress conditions [5], [7]–[10]. In response to severe ER stress, the UPR promotes ER absorption through autophagy and ultimately may induce cell death. ER stress and the UPR have been implicated in many human diseases, including diabetes, inflammatory disease, neurodegenerative disease, secretory cell malignancies, and other cancers [6], [11], [12].

The canonical metazoan UPR is orchestrated by three major ER transmembrane signaling proteins (IRE1, PERK, and ATF6), and three bZIP-family transcription factors (XBP1, ATF4, and cleaved ATF6) (Figure S1A) [2], [5], [6]. The most ancient of these transmembrane proteins, IRE1, is a cytoplasmic endoribonuclease and kinase that senses unfolded proteins in the ER. In response to ER stress, the IRE1 RNAse initiates cytoplasmic splicing of the mRNA encoding XBP1, the transcription factor that is most central to the UPR. The IRE1 kinase contributes to ER homeostasis by regulating the IRE-1 endonuclease activity, and transmits signals through JNK, p38, and other pathways. The kinase PERK phosphorylates the translation initiation factor eIF2α, thereby globally decreasing translation. This reduces the ER protein-folding load, but also favors translation of mRNAs that encode protective proteins, including ATF4. ATF6 resides in the ER membrane but is transported to the Golgi and cleaved in response to ER stress. The activation status of these transmembrane proteins is influenced by their interactions with the ER chaperone BiP (HSP-3/-4 in *C. elegans*).

The ER lumen maintains an oxidative environment, in contrast to the cytoplasm, because the ER enzyme systems that form disulfide bonds generate reactive oxygen species (ROS) [1], [13], [14]. Accordingly, ER stress may eventually lead to cellular oxidative stress and activation of oxidative stress defense genes [15]. Metazoan oxidative and xenobiotic stress responses are orchestrated mainly by the Nrf bZIP-family transcription factors (Nrf1, 2, 3 in mammals). Nrf-family proteins regulate genes involved in various small molecule detoxification processes, including glutathione biosynthesis and conjugation, and have been implicated in longevity assurance in invertebrates and mammals [16]–[21]. These transcription factors have recently been shown to function in proteasome regulation, stem cell maintenance, and metabolism, suggesting that they may control a wider range of processes than previously realized [22]–[26]. It has been reported that mammalian Nrf1 and Nrf3 associate with the ER membrane and nuclear envelope [27]–[30], and that Nrf2 is phosphorylated by PERK [31], [32]. While these last observations are intriguing, it is unknown whether Nrf-family proteins might actually be involved in ER stress defenses, either through mobilizing an oxidative stress response or participating in the UPR itself.

The nematode *C. elegans* has been a valuable system for investigating how Nrf proteins function and are regulated *in vivo*, because of its advantages for employing genetics to elucidate regulatory networks, and performing whole-organism analyses of stress resistance and survival. The *C. elegans* Nrf ortholog SKN-1 plays a critical role in resistance to oxidative and xenobiotic stress, and in various pathways that extend lifespan [16], [17], [19], [23], [33]. Here we describe a comprehensive analysis of whether SKN-1 might be involved in the UPR. We found that under ER stress conditions SKN-1 directly activates many genes involved in ER function, including canonical ER signaling and transcription factors that in turn induce *skn-1* transcription. Importantly, this response is distinct from that which SKN-1 mobilizes under oxidative stress conditions. SKN-1 is required for resistance to ER stress, including reductive stress, a surprising finding given the importance of SKN-1 for oxidative stress defense. Unexpectedly, UPR signaling is needed for SKN-1 to mobilize an oxidative stress response, suggesting that the ER has a licensing and possibly sensing role during oxidative and xenobiotic stress responses.

## Results

### SKN-1 Directly Regulates ER Stress Genes

Several observations led us to investigate whether SKN-1/Nrf might be involved in ER stress defenses. Expression profiling that we performed in *C. elegans* under normal and oxidative stress conditions suggested that SKN-1 regulates a number of genes that are involved in UPR or ER functions [21]. These included *atf-5* (UPR transcription factor ATF4), *ckb-4* (choline kinase), *pcp-2* (prolyl carboxypeptidase), and many genes encoding xenobiotic metabolism enzymes that localize to the smooth ER (Table S1). Moreover, a genome-wide Chromatin Immunoprecipitation (ChIP) analysis of *C. elegans* L1 stage larvae (MOD-ENCODE) [34] detected binding of transgenically expressed SKN-1 at the predicted regulatory regions of numerous genes involved in UPR- or ER processes, including UPR signaling and transcription (*ire-1, xbp-1, pek-1, and atf-6*), Ca^++^ signaling, and protein folding and degradation (Table S1).

**Figure 1:**
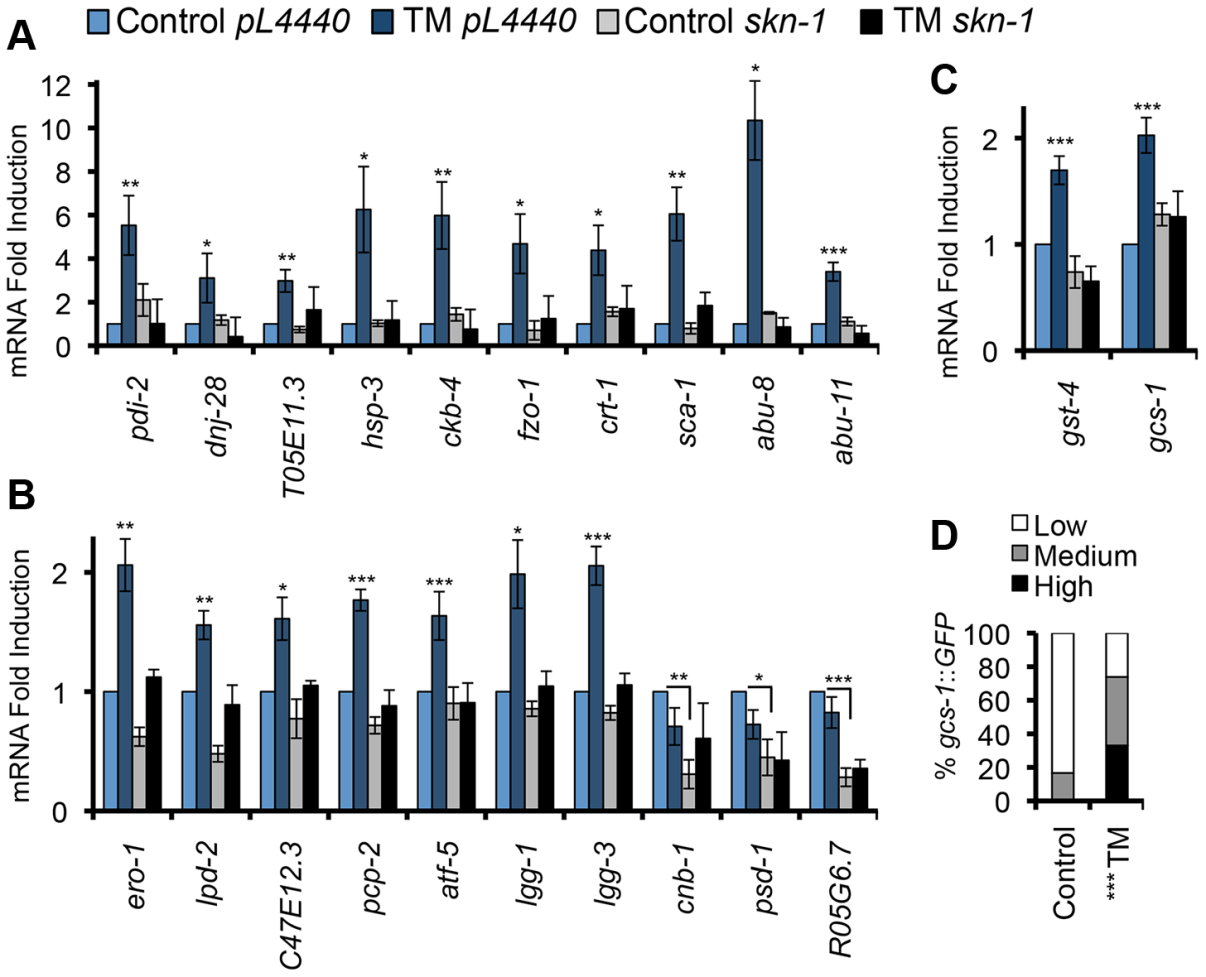
SKN-1 regulates diverse functions in response to ER stress. (A, B) ER stress induces *skn*-1-dependent activation of ER- or UPR-associated genes. qRT-PCR was performed after RNAi Control (*pL4440* in all panels) or *skn-1* RNAi, and Control or 5 µg/ml TM treatment. Known or predicted functions of these genes are described in Table S1. Genes are grouped in (A) or (B) according to the extent of TM-induced activation, and plotted on different scales. All analyses of TM-regulated gene expression involved a 16 hr TM treatment, based upon a time-course experiment (Figure S1B) and published work in *C. elegans* [15]. Shorter time courses were chosen for other ER stress treatments (Figure 4, legend). (C) Upregulation of SKN-1-regulated oxidative stress defense genes in response to TM. Error bars represent SEM, * p≤.05, ** p≤.01, *** p≤.001, relative to *pL4440* Control. All qRT-PCR p-values were calculated as one or two-sided t-test as appropriate with n≥3. (D) Activation of the *gcs-1*::*GFP* transgene in the intestine, with GFP expression scored as High, Medium, or Low. *** p<.0001 chi^2^ method. See Experimental Procedures for scoring method. See also Figure S1 and Table S1.

To investigate whether SKN-1 might be involved in the UPR, we first used quantitative (q) RT-PCR to investigate whether it is needed for expression of representative ER stress-induced or ER maintenance genes, many of which are predicted to be SKN-1 targets (Table S1). In these initial gene expression studies we induced ER stress by treating *C. elegans* with the N-linked glycosylation inhibitor tunicamycin (TM), at a concentration that readily induces the UPR but does not cause detectable toxicity (5 µg/ml, Figure S1B) [15]. TM treatment resulted in *skn-1*-dependent upregulation of numerous canonical or predicted UPR- or ER-related genes (Figures 1A and 1B, Table S1). *skn-1* was also required for the basal expression of *psd-1*, *R05G6.7*, and *cnb-1*, even though these genes were not activated by TM (Figures 1A and 1B). TM-induced ER stress also upregulated two direct SKN-1 targets that are involved in glutathione metabolism (*gcs-1* and *gst-4*) [19] in a *skn-1*–dependent manner, and transgenic reporter analysis detected *gcs-1* activation in the intestine, the *C. elegans* counterpart to the gut, liver, and adipose tissue (Figures 1C and 1D). Importantly, however, ER stress did not activate various other genes that are typically induced by SKN-1 under oxidative stress conditions (Figure S1C). Taken together, the data indicate that SKN-1 mediates a response to ER stress, but also that this response does not correspond simply to its oxidative stress defense function.

**Figure 2:**
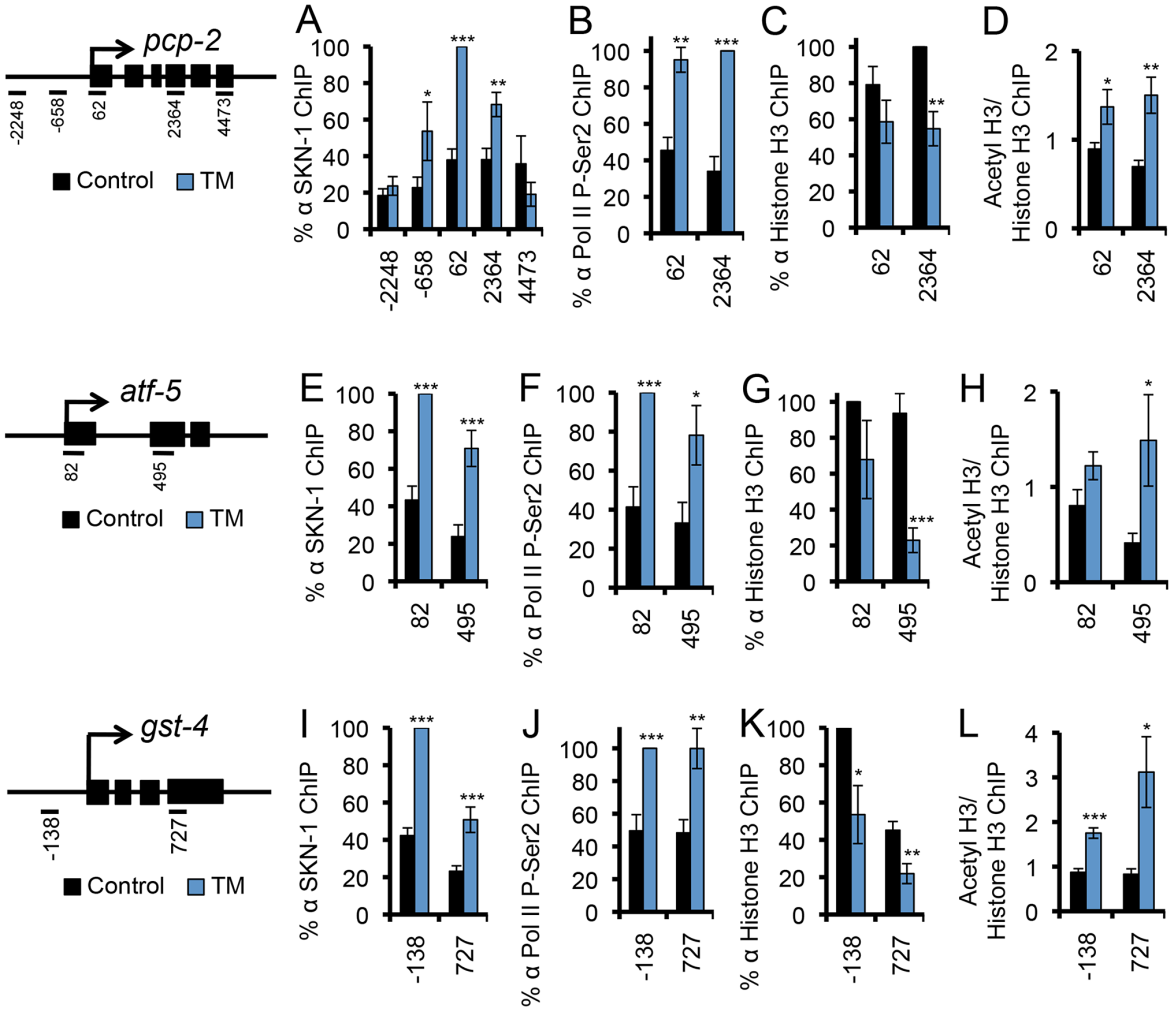
SKN-1 directly regulates target genes during the UPR. (A–L) ER stress-induced SKN-1 recruitment and transcriptional activation was analyzed at the SKN-1-regulated genes *pcp-2* (A–D), *atf-5* (E–H), and *gst-4* (I–L). TM treatment leads to SKN-1 recruitment (A, E, I), accumulation of Pol II that is phosphorylated at CTD Ser 2 (P-Ser2) (B, F, J), decreased Histone H3 occupancy (C, G, K), and increased H3-AcK56 density (D, H, L) at the site of transcription. Maps mark qPCR amplicons relative to the predicted transcription start site, with exons marked as black boxes. % ChIP signal is relative to input, and normalized to the highest signal for each run [44]. In (D, H, L), a ratio of acetyl histone to histone signal is presented. For ChIP experiments in this study error bars represent SEM, and * p≤.05, ** p≤.01, *** p≤.001, relative to *pL4440* Control calculated using one-sided student's t-test. See also Figure S2.

To investigate whether SKN-1 activates genes directly during ER stress, we used ChIP to detect endogenous SKN-1 and markers of transcription activity at *pcp-2*, *atf-5*, and *gst-4*, each of which is flanked by SKN-1 binding sites and upregulated by oxidative and ER stress in a *skn-1*-dependent manner [21] (Figures 1B and 1C). SKN-1 was readily recruited to these genes in response to either TM-induced ER stress or Arsenite (AS)-induced oxidative stress (Figures 2A, 2E, 2I, and S2A-S2C). During transcription, RNA Polymerase II (Pol II) is phosphorylated on Ser 2 of its C-terminal domain (CTD) repeat (P-Ser2) [35]. At each gene we examined, ER stress increased Ser 2 phosphorylation levels (Figures 2B, 2F, and 2J). Also consistent with transcriptional activation, at these loci ER stress increased acetylation of Histone H3, another marker of transcription activity [36], but reduced overall Histone H3 occupancy (Figures 2C, 2D, 2G, 2H, 2K, and 2L). Taken together, our findings suggest that SKN-1 directly activates a major transcriptional response to ER stress.

### Dependence of Core UPR Gene Induction on SKN-1

**Figure 3:**
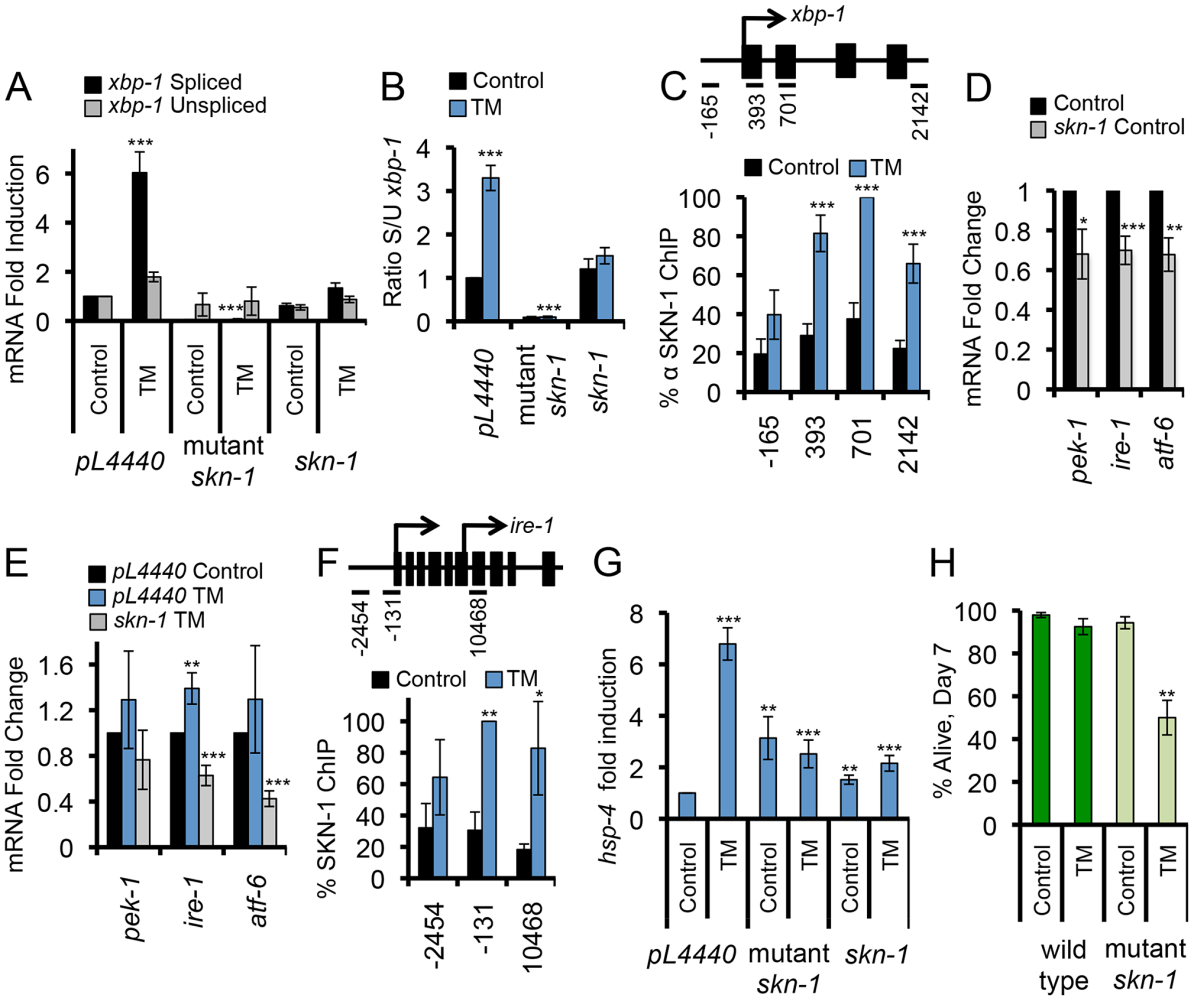
SKN-1 regulates core UPR genes. (A) SKN-1 is required for TM-induced accumulation of spliced *xbp-1* mRNA. Levels of *xbp-1* mRNA forms were analyzed by qRT-PCR with isoform-specific primers, and are presented as the *xbp-1s/xbp-1u* ratio in (B). *skn-1* RNAi and mutant animals were analyzed compared to wild type. *skn-1* refers to *skn-1* RNAi, and *skn-1* mutant refers to the *skn-1(zu67*) allele in all figures unless otherwise indicated. (C) ER stress induces SKN-1 recruitment along the *xbp-1* gene. ChIP analysis is presented as in Figure 2. (D, E) Importance of *skn-1* for expression of core UPR genes under basal (D) and TM-treatment (E) conditions, assayed by qRT-PCR. (F) Binding of SKN-1 to the *ire-1* locus, analyzed by ChIP. (G) SKN-1-dependence of TM-induced *hsp-4/BiP* expression, assayed by qRT-PCR. (H) *skn-1* mutants are sensitized to TM-induced ER stress. Survival of wild type and *skn-1* mutant animals was assayed after 7 days of Control or high-dose TM treatment (35 µg/ml). Error bars represent SEM, and * p≤.05, ** p≤.01, *** p≤.001, relative to *pL4440* Control calculated using student's t-test. See also Figure S3 and Table S2.

We next investigated whether SKN-1 might regulate expression of core UPR signaling and transcription factors, as predicted by the MOD-ENCODE data [34]. XBP-1 is central to the UPR, and in mammals it controls transcription of other core UPR genes (*atf4/atf-5*, and *BiP/hsp-4*) along with many downstream genes [6], [37]. During the UPR, *xbp-1* expression is regulated at the level of transcription, as well as through cytoplasmic splicing of its mRNA by the IRE-1 endoribonuclease (Figure S1A) [5], [6]. The spliced form of the *xbp-1* mRNA (*xbp-1s*) encodes the transcriptionally active form of XBP-1 (XBP-1s). When SKN-1 was lacking, ER stress failed to induce accumulation of each *xbp-1* mRNA form and, remarkably, decreased the ratio of *xbp-1s* to the unspliced *xbp-1* form (*xbp-1u*) (Figures 3A, 3B, and S3A). The *xbp-1* locus includes a predicted SKN-1 binding site (not shown), and ChIP results indicated that endogenous SKN-1 accumulates at the *xbp-1* site of transcription in response to ER stress (Figure 3C). This evidence that SKN-1 directly regulates *xbp-1* could account for the reduction in total *xbp-1* mRNA, but not the apparent effect of SKN-1 on *xbp-1* splicing. A plausible explanation is that lack of SKN-1 also reduced basal and ER stress-induced expression of *ire-1* (Figures 3D and 3E). Moreover, we observed that SKN-1 is recruited to the *ire-1* locus in response to ER stress (Figure 3F), consistent with MOD-ENCODE evidence that *ire-1* may be a SKN-1 target [34].

SKN-1 was also required for expression of other core UPR genes. Mutation or RNAi knockdown of *skn-1* prevented ER stress-induced expression of the unfolded protein chaperone and sensor HSP-4 (BiP) (Figure S1A)(Figures 3G, S3B, and S3C). Binding of SKN-1 at *hsp-4* was not detected in the MOD-ENCODE study of L1 larvae [34], but our ChIP evidence indicated that both SKN-1 and XBP-1 bind directly to the *hsp-4* locus (Figures S3D and S3E), which includes predicted SKN-1 binding sites (not shown). SKN-1 similarly contributed to expression of the core UPR factors *pek-1* and *atf-6* (Figures 3D and 3E). Our evidence that SKN-1 is important for transcriptional induction of core UPR signaling and regulatory factors predicts that it should be important for *C. elegans* survival under ER stress conditions. Treatment with TM at a 7-fold higher concentration (35 µg/ml) than is sufficient to induce the UPR impaired the survival of *skn-1* mutants but not wild type animals (Figure 3H and Table S2). We conclude that SKN-1 plays a critical role in the UPR through its direct transcriptional regulation of core UPR factors, along with many downstream genes.

### Activation of SKN-1 by ER Stress Independently of Oxidative Stress

**Figure 4:**
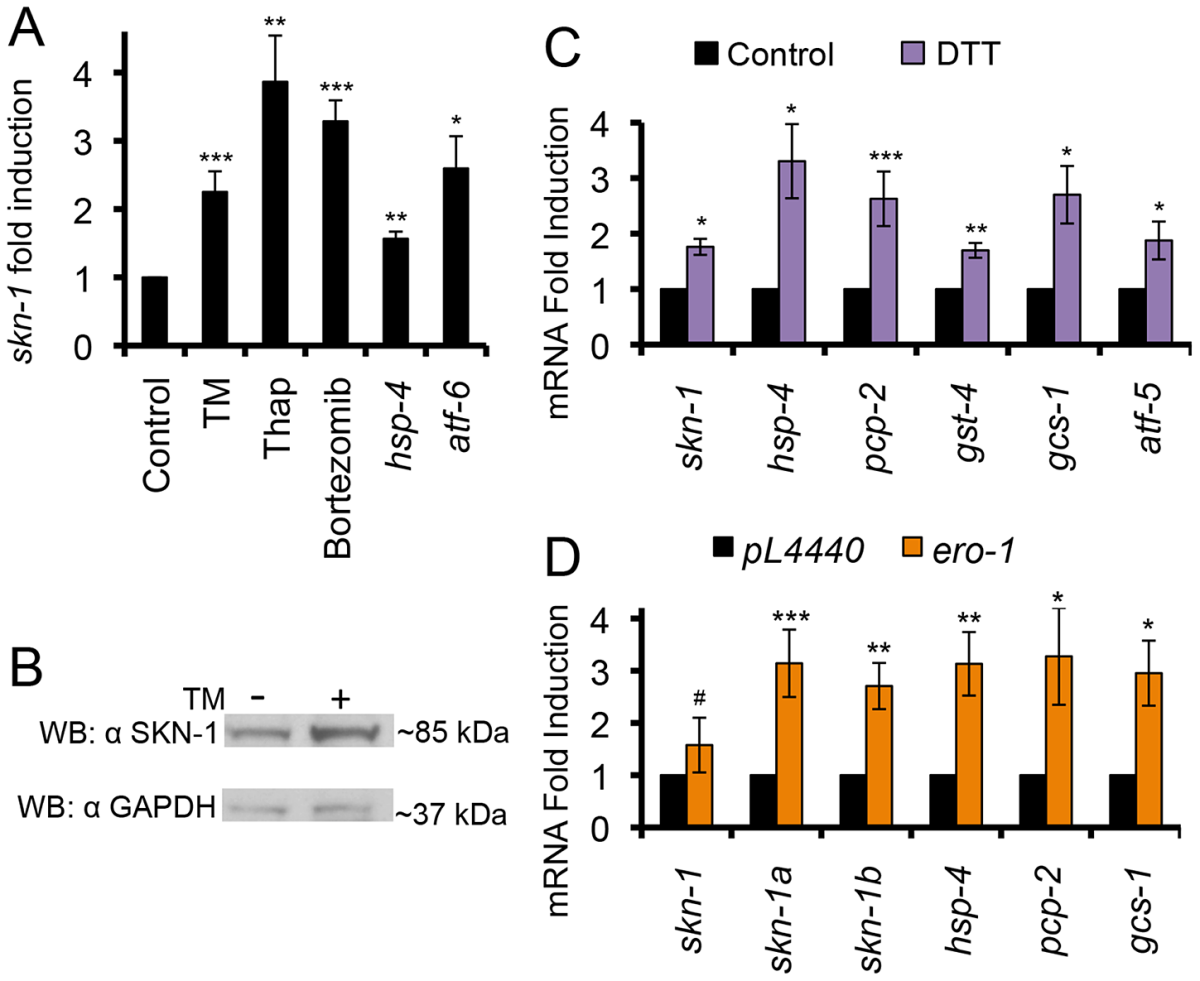
ER stress activates SKN-1 independently of oxidative stress. (A) Treatment with TM (16 hrs), thapsigargin (Thap, 2 hrs), or bortezomib (6 hrs) increased *skn-1* mRNA levels, as determined by qRT-PCR. RNAi knockdown of *hsp-4* or *atf-6* also increased *skn-1* mRNA levels. (B) Increased endogenous SKN-1 protein levels in response to TM-induced ER stress. SKN-1 was detected by Western blotting with the polyclonal antibody, with GAPDH serving as the loading control. (C) Induction of *skn-1* expression and SKN-1-regulated UPR target genes by reductive ER stress (DTT treatment for 2 hrs), assayed by qRT-PCR. (D) Induction of the UPR, *skn-1* expression, and SKN-1 target genes by *ero-1* RNAi, assayed by qRT-PCR. Different primer sets were used to distinguish among mRNAs that correspond to different *skn-1* isoforms. Error bars represent SEM, and * p≤.05, ** p≤.01, *** p≤.001, relative to *pL4440* Control calculated using student's t-test. See also Figure S4 and Table S3.

We next examined whether expression of *skn-1* itself is increased when the ER becomes stressed, and whether various conditions that cause ER stress affect SKN-1 activity. Treatment with TM increased the levels of multiple mRNA species that encode SKN-1 isoforms (Figure 4A and S4A). In addition, non-lethal treatment with either the Ca^++^ pump inhibitor thapsigargin (Thap) or the proteasome inhibitor Bortezomib upregulated transcription of *skn-1*, and various SKN-1-regulated genes (Figures 1, 4A, and S4B–S4C). Finally, knockdown of either the ER chaperone *hsp-4* or the UPR transcription factor *atf-6* resulted in transcriptional upregulation of *skn-1* and many of its ER stress targets in the absence of drug treatment, presumably because of an elevated level of ER stress (Figures 4A, S4D and S4E). We conclude that *skn-1* transcription and activity are increased in response to a variety of conditions that are associated with ER stress.

An important hallmark of the UPR is a decrease in the overall levels of translation [5], [6]. This relieves stress on the ER, and allows translation of *atf4* and other protective genes to be maintained or even increased. We investigated whether SKN-1 translation is similarly “spared” under ER stress conditions. Supporting this idea, TM treatment increased SKN-1 protein levels, a trend that was observed in Western and IP-Western analyses of whole animals with two specific SKN-1 antibodies (Figures 4B and S4F–S4I). Based upon its size, this approximately 85 kD SKN-1 species is likely to represent SKN-1a, the largest SKN-1 isoform. While this size is larger than the expected SKN-1a MW of 70 kD, SKN-1 is phosphorylated and predicted to be glycosylated, as is characteristic of Nrf1 and Nrf3 (not shown) [17], [28], [38]–[40]. Our finding that SKN-1 protein levels are increased by ER stress is consistent with earlier evidence that SKN-1 translation seemed to be preserved when translation initiation was inhibited [41].

Prolonged ER stress leads to accumulation of reactive oxygen species (ROS) and induction of an oxidative stress response [15], [42], making it important to determine whether ER stress treatments might activate SKN-1 simply through a secondary response to oxidative stress. Arguing against this interpretation, even though SKN-1 is well known to defend against oxidative stress, we found that *reductive* ER stress also induced a SKN-1-dependent response. The reducing agent dithiothreitol (DTT) initiates the UPR through reduction of Cys-Cys bonds in the ER [43]. DTT treatment resulted in transcriptional induction of *skn-1* and many of its target genes, and increased SKN-1 protein levels (Figures 4C and S4J). SKN-1 appeared to be required for its downstream targets to be activated by DTT-induced reductive stress (Fig. S4K), and knockdown of either *skn-1* or *hsp-4* rendered *C. elegans* comparably sensitive to reductive stress from DTT (Figure S4L and Table S3). Another way to reduce oxidation in the ER is through inhibiting expression of the oxidase ERO-1, which promotes Cys-Cys crosslinking [43]. *ero-1* RNAi decreases ROS levels, initiates the UPR, and extends lifespan [15]. As observed with DTT, *ero-1* RNAi transcriptionally activated *skn-1* and several of its downstream targets (Figure 4D).

Additional lines of evidence support the idea that SKN-1 acts in the UPR independently of its role in oxidative stress defense. Many genes that are activated by SKN-1 under oxidative stress conditions were not upregulated by ER stress (Figures S1C and S4M). Oxidative stress from AS treatment induced the SKN-1::GFP (green fluorescent protein) fusion to accumulate to high levels in intestinal nuclei, as previously described (Inoue, et al., 2005), but this did not occur in response to ER stress (Figure S4N). Finally, we did not observe increased levels of oxidized proteins under conditions of TM-induced ER stress (Figure S4O). Taken together, the data show that ER stress directs SKN-1 to activate a specific set of its target genes independently of any secondary oxidative stress response.

### Regulation of SKN-1 by UPR Factors

**Figure 5:**
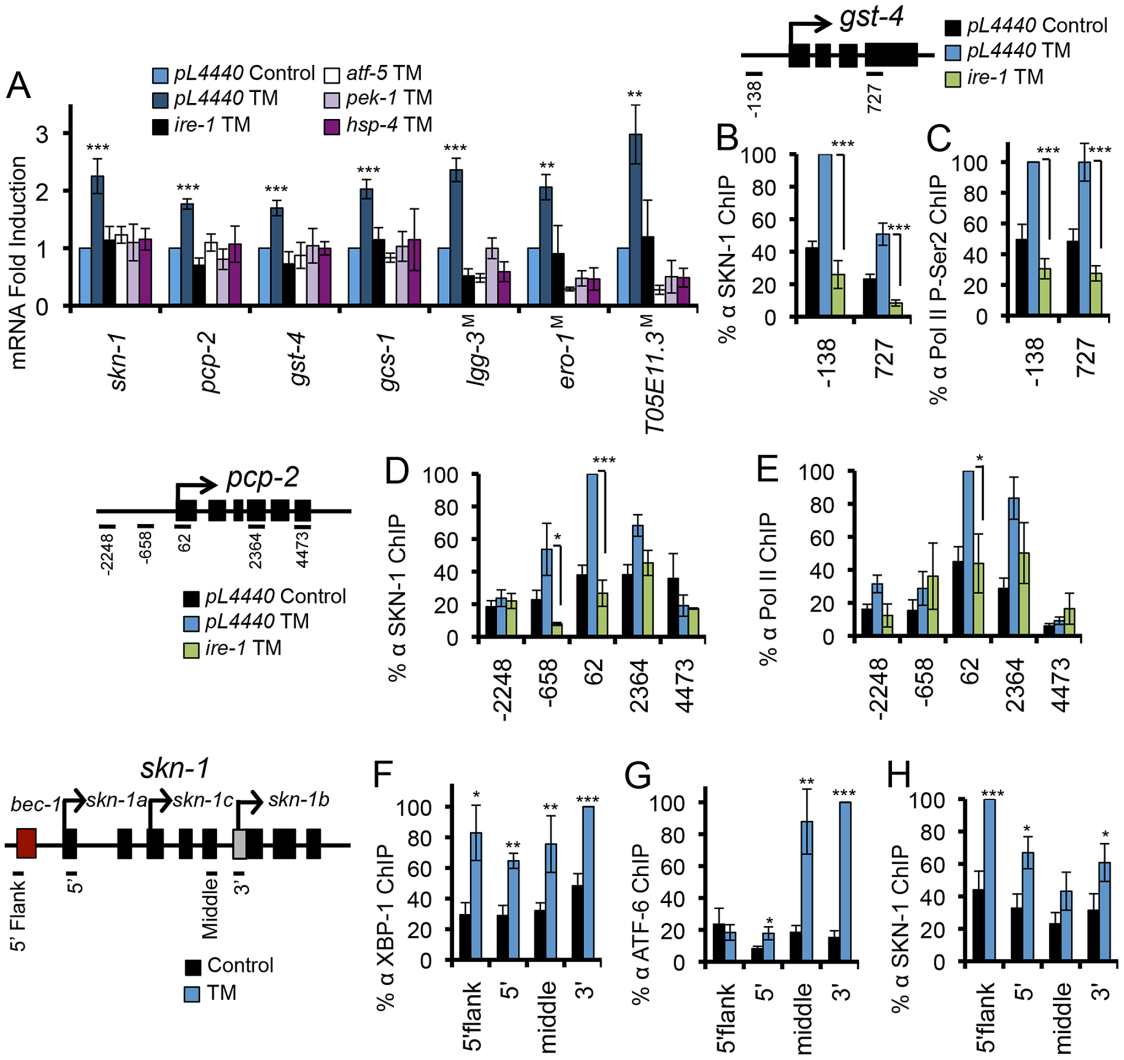
UPR factors required for ER stress-induced SKN-1 activation. (A) ER stress-induced activation of *skn-1* and its target genes requires core UPR factors. RNA levels were assayed by qRT-PCR after RNAi against core UPR genes or in core UPR factor mutants (indicated by ^M^) after TM treatment. (B-E) IRE-1 is required for ER stress-induced SKN-1 accumulation and activity at SKN-1 target genes *gst-4* and *pcp-2*. Presence of SKN-1 and transcription markers was assayed by ChIP as in Figure 2, and *ire-1* was knocked down by RNAi. (F–H) Endogenous XBP-1 (F), ATF-6 (G), and SKN-1 (H) bind within the *skn-1* gene locus in response to TM-induced ER stress, with binding assayed by ChIP. Multiple start sites are noted within the *skn-1* locus. Error bars represent SEM, and * p≤.05, ** p≤.01, *** p≤.001 by student's t-test, relative to *pL4440* Control unless otherwise indicated. See also Figure S5.

If ER signaling pathways regulate SKN-1, then key UPR signaling and transcription factors should be required for ER stress to activate SKN-1 and its target genes. Accordingly, RNAi or mutation of *ire-1*, *atf-5*, *pek-1*, or *hsp-4* essentially prevented ER stress from inducing transcription of *skn-1* and several of its target genes (Figure 5A). Knockdown of *xbp-1* under control conditions increased background expression of some SKN-1 isoforms and target genes (*skn-1b*, *pcp-2*, *gst-4*, *hsp-4*), possibly because ER stress was increased, but also interfered with ER stress-induced activation of several of these genes (*skn-1a*, *pcp-2*, *gcs-1*, *hsp-4*) (Figure S5A). RNAi against *ire-1*, which is essential for XBP-1s expression [5], [6], also blocked TM-induced accumulation of SKN-1, Pol II, or P-Ser2 at the *gst-4*, *pcp-2*, and *atf-5* loci (Figures 5B–5E, S5B and S5C). Knockdown of *hsp-4* or *pek-1* had a similar effect (Figure S5D–S5G). The evidence indicates that, in general, core UPR factors are required for ER stress to upregulate expression of SKN-1 and its target genes.

The most straightforward mechanism through which ER stress could increase *skn-1* transcription is through the direct regulation of *skn-1* by one or more of the canonical UPR transcription factors. During the UPR, downstream gene transcription is controlled largely by XBP1 and ATF4, which may regulate each other directly, with ATF-6 playing a more specialized role [8], [15], [37]. The *skn-1* locus contains possible XBP-1 and ATF-6/XBP-1 binding elements (not shown), and genome-wide ChIP studies suggest that mammalian Nrf3 may be a direct XBP1 target [37]. We determined that XBP-1 binds within the *skn-1* locus in response to ER stress, suggesting direct regulation (Figure 5F), a remarkable parallel to the direct regulation of *xbp-1* by SKN-1 (Figure 3C). Moreover, ATF-6 was also recruited to the *skn-1* locus in response to ER stress (Figure 5G). In mammals, XBP-1 may regulate its own expression [37]. Our ChIP analysis indicated that SKN-1 also binds to its own locus with ER stress (Figure 5H), suggesting that SKN-1, XBP-1, and ATF-6 together regulate *skn-1* transcription. ER stress also resulted in XBP-1 and ATF-6 recruitment to the direct SKN-1 targets *pcp-2* and *gst-4* (Figures S5H–S5K). Together, the evidence suggests that SKN-1, XBP-1, and ATF-6 may function together to regulate several downstream genes. We conclude that SKN-1 is transcriptionally integrated into the UPR, in which it functions upstream, downstream, and in parallel to the known core UPR transcription factors.

**Figure 6:**
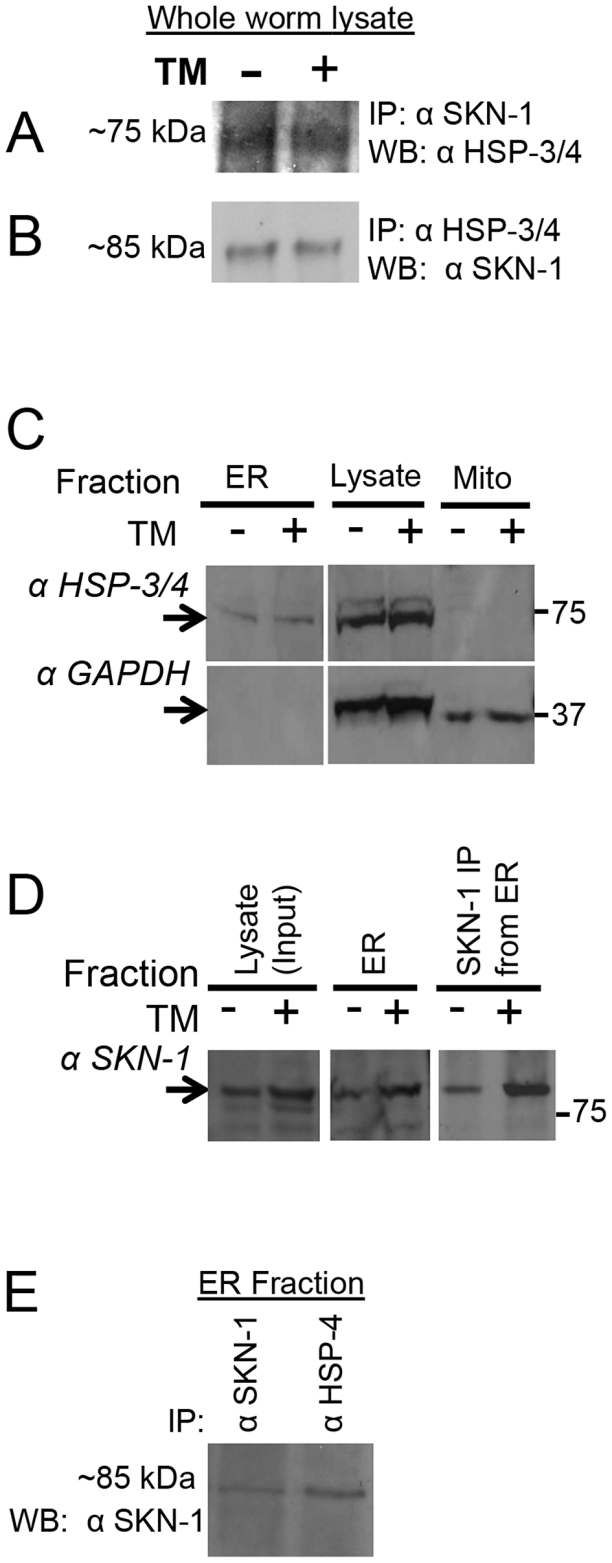
Association of SKN-1 with the ER. (A, B) Interaction between endogenous SKN-1 and HSP-3/4, detected by IP/Western. Lysates were prepared from animals in which proteins had been crosslinked under ChIP conditions. (A) Monoclonal αSKN-1 IP blotted with αHsc3 (HSP-3/4). (B) αHsc3 (HSP-3/4) IP blotted with monoclonal αSKN-1. (C–E) Analyses of ER fractions prepared from whole worms. The fractionation scheme is described in Fig. S6B. (C) Detection of endogenous HSP-3/4 and the cytoplasmic marker GAPDH in ER and Mitochondrial fractions, and total worm lysate. Note the enrichment of the ER marker HSP-3/4 compared to GAPDH in the ER fraction. TM indicates lysates from animals that had been treated with TM. (D) Presence of endogenous SKN-1 in the ER fraction, detected by western and IP/western blotting. Note that TM treatment increased the levels of SKN-1 protein. (E) Association between endogenous SKN-1 and HSP-3/4 within the ER fraction, detected with polyclonal αSKN-1 and αBiP (HSP-3/4), by IP/Western that was performed without crosslinking. Fractionations and analyses were performed independently twice, with similar results. See also Figure S6.

The mammalian SKN-1 orthologs Nrf1 and Nrf3 have been detected in association with the ER (see Introduction), raising the question of whether this might also be true for a proportion of SKN-1. Consistent with this idea, Nrf1 and the SKN-1a isoform each contain a predicted transmembrane domain [27] (Figure S6A). To investigate whether SKN-1 might be present at the ER, we asked whether it might be detected in association with the ER-resident chaperone BiP (HSP-3/-4)(Figure S1A). We performed co-immunoprecipitation (IP) analyses of intact worms that had been crosslinked with formaldehyde as in our ChIP experiments. These conditions capture direct and indirect *in vivo* interactions that occur within approximately 2 Å, and allow for high-stringency detergent and salt-based washings that minimize non-specific binding [44], [45]. Under both normal and ER stress conditions, association between HSP-4 and SKN-1 was readily detected by high-stringency IP performed in either direction (Figure 6A and 6B). As in Figure 4B, the size of this SKN-1 species suggested that it may correspond to SKN-1a. The data suggest that some SKN-1 may be produced at the ER and might remain associated with this organelle.

Given that BiP has been found in other cellular locations besides the ER [46], we also investigated whether SKN-1 is present in a cellular fraction that is enriched for the ER (Figure S6B). SKN-1 was readily detectable in an ER fraction that included HSP-4, but not the cytoplasmic protein GAPDH (Figures 6C and 6D). The interaction between endogenous SKN-1 and HSP-4 was confirmed within this ER fraction by a co-IP that was performed without crosslinking (Figure 6E). Together, our findings suggest that the association of SKN-1/Nrf proteins with the ER is evolutionarily conserved.

### SKN-1-Mediated Oxidative Stress Responses Depend upon ER Signaling

**Figure 7:**
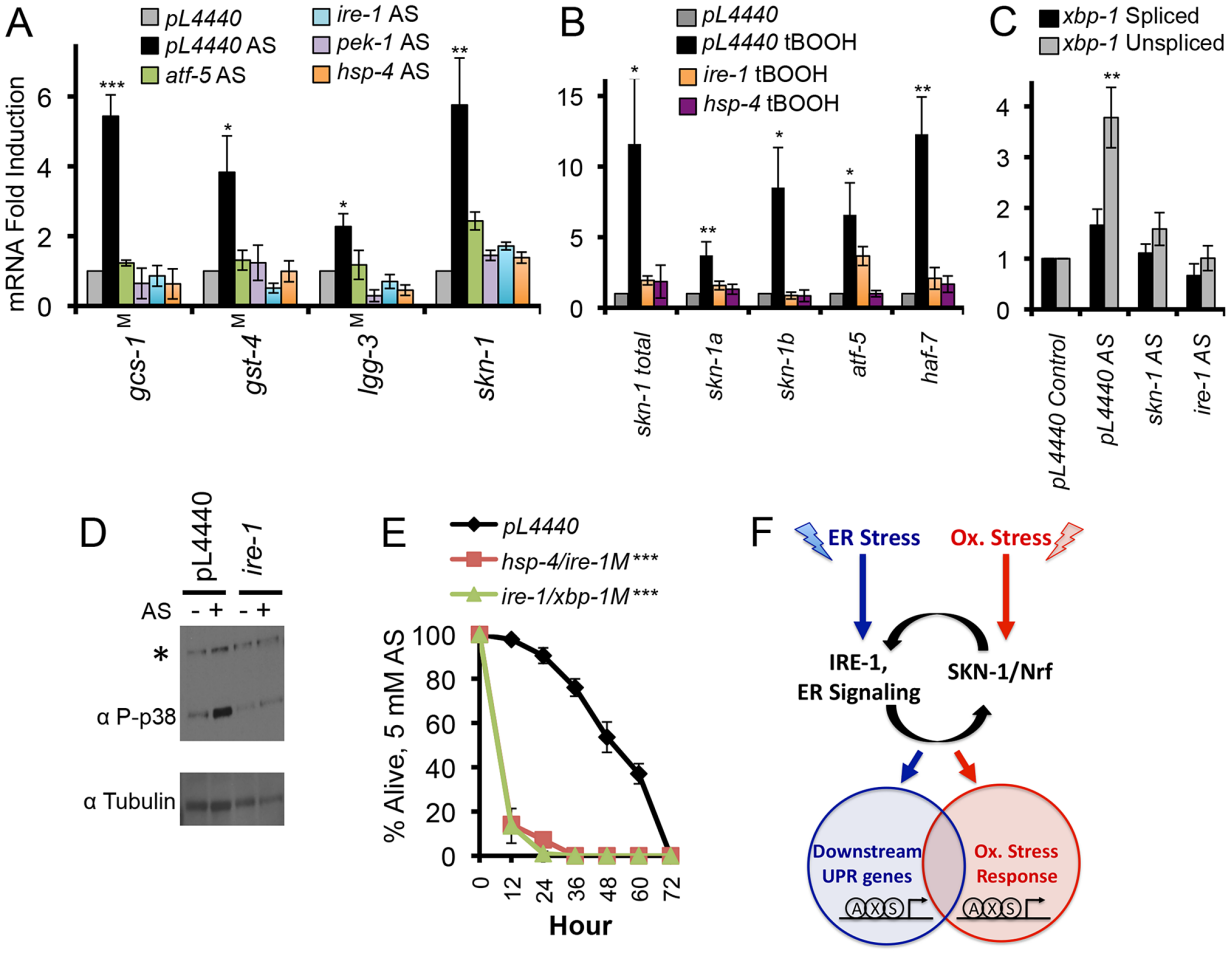
Dependence of oxidative stress responses on UPR components. (A, B) Importance of core UPR genes for SKN-1-mediated oxidative stress responses. Induction of *skn-1* and *skn-1* target gene transcription by AS (A) or t-BOOH (B) was impaired by RNAi against core UPR genes or in core UPR factor mutants (indicated by ^M^). qRT-PCR was performed after treatment with 5 mM AS for 1 hour, or 12 mM t-BOOH for 1 hour. (C) Accumulation of *xbp-1* mRNA in response to AS-induced oxidative stress. Note the predominant increase in the unspliced form. (D) Dependence of AS-induced p38 phosphorylation on *ire-1*. Phosphorylated (active) p38 was assayed by phospho-specific antibody as in [38], and *ire-1* expression was knocked down by RNAi. *background signal. (E) UPR factors are required for oxidative stress defense. Survival of AS treatment (5 mM) was scored in RNAi Control, *hsp-4(RNAi)/ire-1(zc14)*, and *ire-1(RNAi)/xbp-1(SJ17)* animals (M indicates mutant). Error bars represent SEM, and * p≤.05, ** p≤.01, *** p≤.001, relative to *pL4440* Control calculated using student's t-test. (F) Functional integration of the ER and oxidative stress responses through SKN-1 and canonical UPR components (see text). SKN-1 is essential for the UPR because it directly controls transcription of most UPR signaling and transcription factors. These UPR factors in turn regulate SKN-1 expression, and function in concert with SKN-1 at downstream targets. This is shown arbitrarily as SKN-1 (S) binding to target promoters together with XBP-1 (X) and ATF- 6 (A). SKN-1 and mammalian Nrf proteins are present in the ER, suggesting a possible signaling role. UPR factors are required not only for SKN-1 to function in the context of the UPR, but also for SKN-1 to mobilize distinct oxidative stress responses. See also Figure S7 and Table S4.

Our finding that UPR factors are required for SKN-1 activity to be increased under ER stress conditions raised a related question: might UPR-related mechanisms also be involved in SKN-1 responses to oxidative stress? Surprisingly, we found that RNAi or mutation of core UPR signaling and transcription factors (*atf-5*, *pek-1*, *ire-1*, *hsp-4* and *xbp-1*) impaired oxidative stress (AS)-induced activation of several SKN-1 target genes, including *skn-1* itself (Figures 7A, 7C, and S7A). Similarly, *ire-1* RNAi attenuated activation of the *gcs-1::GFP* reporter in the intestine (Figure S7B). This impairment of the oxidative stress response is particularly striking because *ire-1* RNAi actually increased oxidized protein levels, in contrast to the mild AS treatment conditions used for gene expression analyses (Figure S4O).

Importantly, oxidative stress from AS did not simply activate the canonical UPR. Many SKN-1-regulated genes that were induced by oxidative stress were not upregulated by ER stress, and vice-versa (Figures S1C, S4M, and S7C). This shows that SKN-1 mobilizes distinct transcriptional responses to oxidative and ER stress, even if these responses overlap to an extent. Moreover, AS primarily increased accumulation of the *unspliced xbp-1* mRNA form (*xbp-1u*), in striking contrast to the increase in *xbp-1s* levels that is characteristic of ER stress (Figures 3A and 7C). Treatment with the oxidative stressor tert-butyl hydrogen peroxide (tBOOH) induces a SKN-1-dependent response that overlaps with the AS response, but includes SKN-1-independent activation of many genes that are otherwise SKN-1-dependent [21]. Knockdown of *ire-1* or *hsp-4* inhibited tBOOH from upregulating *skn-1* and some SKN-1 targets (Figure 7B), but did not eliminate activation of other genes (*gcs-1*, *sdz-8*, and *gst-10*; not shown). The data suggest that core UPR factors are needed for SKN-1 to function properly under oxidative stress conditions, in addition to the setting of ER stress.

The extensive regulatory integration that exists among UPR transcription factors, as described by others and in this study (Figures 7A, 7B, and S7A) [8], [15], [37], could explain why multiple UPR-associated signaling and transcription factors are needed for *skn-1* expression to be increased in response to oxidative stress. However, we considered that the UPR might also influence SKN-1 regulation at a post-translational level. In the *C. elegans* intestine SKN-1 is predominantly cytoplasmic under normal conditions, but accumulates in nuclei in response to oxidative stress from AS treatment [38]. This nuclear accumulation was dramatically reduced in animals that had been exposed to *ire-1* RNAi (Figure S7D). The presence of SKN-1 in intestinal nuclei is dependent upon its phosphorylation by the p38 kinase, which is activated by oxidative stress [23], [38], [47]. The IRE-1 kinase activity transmits signals through the JNK and p38 MAPK pathways [6], [48]–[50], and we determined that *ire-1* knockdown largely prevented the increase in p38 signaling that occurs in response to oxidative stress (Figures 7D and S7D). Taken together, these data suggest that IRE-1 is required for oxidative stress to activate SKN-1 post-translationally.

If UPR signaling and transcription factors are required for SKN-1 to mobilize appropriate oxidative stress responses, then oxidative stress sensitivity should be increased when these canonical UPR factors are lacking. Accordingly, RNAi or mutation of these genes significantly increased sensitivity to oxidative stress from exposure to AS, paraquat, or t-BOOH (Figures 7E, S7E, and S7F; Table S4). We conclude that signaling from the ER is required for SKN-1 to respond to oxidative stress, and therefore that UPR-mediated regulation of SKN-1 plays a central role in the homeostatic integration of ER and oxidative stress responses.

## Discussion

### SKN-1 Is a Critical UPR Factor

It is well-established that the canonical UPR transcription factors XBP1, ATF4, and ATF6 control overlapping sets of downstream genes and processes [5], [6], but much less is known about how their responses to ER stress might be integrated with other mechanisms that maintain cellular stress defense and homeostasis. We have determined that the oxidative/xenobiotic stress response regulator SKN-1/Nrf functions as a fourth major UPR transcription factor in *C. elegans*. Without SKN-1, ER stress failed to increase the expression of core UPR signaling and transcription factors, many of which are regulated directly by SKN-1 (*ire-1*, *xbp-1*, *atf-5*, and *hsp-4*; Figures 1, 2, 3 and S3). It was particularly striking that SKN-1 was disproportionally required for production of spliced *xbp-1* mRNA (*xbp-1s*), presumably because of its importance for IRE-1 expression (Figures 3D–F). SKN-1 was also needed for ER stress to upregulate numerous genes that are known or predicted to be involved in various ER- or UPR-related processes, including ER homeostasis (*ero-1, pdi-2*), chaperone-mediated protein folding (*hsp-3*, *hsp-4*, *dnj-28*, *T05E11.3* (HSP-90/GRP94)), autophagy (*lgg-1, lgg-3*), calcium homeostasis (*sca-1, crt-1*), ER membrane integrity (*ckb-4*), and a pathway that defends against ER stress when the canonical UPR is blocked (*abu-8, abu-11* [51]) (Figure 1, 3G and Table S1). Together, our data indicate that SKN-1 regulates transcription of essentially the entire core UPR apparatus and many downstream ER stress defense genes *in vivo*.

We were surprised to find that SKN-1 was so broadly important for UPR transcription events. A trivial explanation for our findings would be that *skn-1* mutants did not need to induce the UPR robustly because they were resistant to ER stress. This explanation was ruled out, however, by our finding that *skn-1* mutants are actually sensitized to ER stress from diverse sources (Figures 3H and S4L). Importantly, our ChIP studies and MOD-ENCODE data [34] indicate that SKN-1 controls many core and downstream UPR genes directly by binding to their promoters (Figures 2, 3, and S3E, Table S1). We also found that ER stress induces SKN-1, XBP-1, and ATF-6 to bind promoters directly to regulate many of the same genes, including *skn-1* itself (Figures 5, S3, and S5). In addition, under ER stress conditions, UPR signaling increased levels of *skn-1* mRNA and protein (Figures 4 and S4), indicating that SKN-1 is controlled by the UPR and is an active participant in this response. Together, our data reveal that a remarkable degree of regulatory and functional integration exists between SKN-1 and the three canonical UPR transcription factors (Figures 7F and S1A).

Although ER stress increases *skn-1*-dependent transcription and SKN-1 occupancy at several downstream gene promoters, it did not detectably alter the overall levels of SKN-1 in intestinal nuclei, at least as indicated by levels of a transgenic GFP fusion protein (Figure S4N). While this might seem paradoxical, we observed a similar situation with reduced TORC1 signaling [19]. Under conditions of low TORC1 activity SKN-1 target genes were activated in a *skn-1*-dependent manner, and this was accompanied by increased SKN-1 binding to their promoters, but not by an obvious increase in the bulk levels of SKN-1 in nuclei. Our finding that SKN-1 binds to downstream UPR genes together with other UPR transcription factors suggests a paradigm that could explain this phenomenon. If SKN-1 binds cooperatively with UPR factors or other co-regulators to some of its targets, this could shift the binding equilibrium to allow those targets to be activated by SKN-1 that is already present in the nucleus, without it being necessary to “flood” the nucleus with higher levels of SKN-1. This scheme might be important for fine-tuning of SKN-1 downstream functions, and for allowing SKN-1 to activate different targets in different situations, as we have observed in this study.

In performing these analyses, we were mindful of the concern that the involvement of SKN-1 in the UPR might derive from its possible role in a secondary oxidative stress response. Several lines of evidence argued against this interpretation. For example, the direct involvement of SKN-1 in regulating multiple core UPR signaling and transcription factors during the UPR (Figures 3 and S3) is not consistent with its UPR functions deriving simply from a secondary oxidative stress response. Moreover, under our ER stress conditions SKN-1 was required for accumulation of the spliced form of the *xbp-1* mRNA, whereas oxidative stress increased levels of the unspliced *xbp-1* message (Figures 3A, 3B, and 7C). It was particularly striking that SKN-1 defended against reductive ER stresses (Figures 4C, 4D, S4J, S4K, and S4L), given the extensively described role of SKN-1/Nrf proteins in oxidative stress responses. These last observations indicated that SKN-1 defends against ER stress *per se*, and not only against oxidative conditions. Importantly, ER stress and the UPR directed SKN-1 to activate some of its target genes that are induced by oxidative stress, but not others (Figure S1C and S4M). On the other hand, many genes that SKN-1 activated under ER stress conditions were not induced by oxidative stress (Figure S7C). Taken together, the data show that SKN-1 does not simply activate oxidative stress defenses in the context of ER stress, but orchestrates a specific transcriptional ER stress response that is integrated into the broader UPR.

Our finding that SKN-1 mobilizes overlapping but distinct responses to ER and oxidative stress defines a new function for this surprisingly versatile transcription factor. It also supports our model that SKN-1/Nrf proteins do not control the same genes under all circumstances, but instead induce protective responses that are customized to the challenge at hand [19], [26]. The idea that SKN-1 works together with canonical UPR transcription factors at downstream genes may provide a model for understanding how particular SKN-1 functions can be mobilized under different conditions, if these proteins and other SKN-1 “partners” guide its activities.

Consistent with reports that Nrf1 and Nrf3 are present at the ER [27]–[30], we found that some SKN-1 also localizes to the ER. We detected association between SKN-1 and the ER chaperone HSP-3/4 (BiP) in crosslinking analyses of intact animals, the presence of SKN-1 within an ER fraction, and association between SKN-1 and HSP-3/4 within that fraction (Figure 6 and S6). Each of these experiments involved analysis of endogenous proteins. These strategies would have detected either direct or indirect interactions, so they do not demonstrate that SKN-1 binds directly to HSP-3/4 (BiP), but they do show that these proteins reside very close to each other at the ER. Apparently, association between SKN-1/Nrf proteins and the ER is evolutionarily conserved. The example of ATF-6, which is activated through cleavage in the Golgi (Figure S1A), predicts that ER-associated SKN-1 might have a signaling function in which it is cleaved in response to ER stress. However, the relative instability of SKN-1 and the presence of smaller isoforms have so far confounded the resolution of this question (not shown). We recently determined that some SKN-1 also localizes to mitochondria and that SKN-1 can promote a starvation-like state when overexpressed, a function that also appears to be conserved in Nrf proteins [26]. Given the extensive communication between the ER and mitochondria [4], [52], our results suggest that SKN-1/Nrf might respond directly to the status of each of these organelles. Consistent with this notion, SKN-1 is required for expression of the *C. elegans* ortholog of mitofusin (*fzo-1*) (Figure 1A), which mediates mitochondrial fusion and mitochondria-ER interactions [4].

Taken together, our findings show that processes controlled by SKN-1/Nrf proteins are critical for ER stress defense and homeostasis, and that SKN-1 is extensively intertwined with the UPR *in vivo*. While differences could exist between *C. elegans* and mammals with respect to regulatory networks, the extent of the functional interactions we have observed predicts that mammalian Nrf proteins are likely to play an important role in the UPR that is distinct from their familiar function in oxidative stress responses.

### Regulation of Oxidative Stress Responses by the UPR

Perhaps our most surprising finding was that core UPR signaling and transcription factors were required for SKN-1 to mount a transcriptional response to oxidative stress (Figures 7 and S7). Cooperative interactions between SKN-1 and UPR transcription factors could account for some of these findings, through their effects on SKN-1 expression, but it was striking that *ire-1* was needed for AS to induce SKN-1 nuclear accumulation, a phenomenon that does not occur under ER stress conditions (Figures S4N and S7D). Moreover, *ire-1* was required for the AS-induced p38 signal that is needed for SKN-1 to be present in nuclei (Figure 7D). These last findings indicate that IRE-1 affects the oxidative stress response at a step upstream of SKN-1. One speculative possibility for further investigation is that the IRE-1 kinase activity might be needed to initiate the oxidative stress-induced p38 signal. Together, our data show that signaling from the ER is required to “license” the oxidative/xenobiotic stress response, and suggest that the ER might function in effect as a stress sensor. This importance of the UPR for SKN-1 activity may have implications for our understanding of aging and longevity assurance. SKN-1/Nrf not only defends against resistance to various stresses, but is also important in pathways that affect longevity, including insulin-like, TORC1, and TORC2 signaling, and dietary restriction [16], [17], [19], [20]. IRE-1 and XBP-1 have each been implicated in longevity [53], [54], making it important to determine the extent to which these UPR-based mechanisms might influence aging through regulation of SKN-1/Nrf and its functions.

Why would such extensive integration have arisen, in which SKN-1/Nrf is essential for the UPR, and signaling from the ER is needed for SKN-1/Nrf activities that are distinct from the UPR (Figure 7F)? SKN-1/Nrf controls cellular processes that profoundly influence the ER. Its target genes drive synthesis of glutathione, the major redox buffer within the ER, and encode many endobiotic and xenobiotic metabolism enzymes that reside on or within the smooth ER (Table S1) [20], [21], [55]. Under some circumstances SKN-1/Nrf also regulates proteasome expression and activity, and numerous chaperone genes [20], [21], [23]–[25]. One possibility is that the influence of SKN-1 could attune the UPR to events taking place in the cytoplasm. It might be advantageous to mount a robust transcriptional UPR if the cytoplasm is under duress, for example, and to moderate the UPR when cytoplasmic stress is low. Under these conditions, SKN-1 activity would be relatively high and low, respectively. SKN-1 activity is also comparatively low when translation rates are high [19], [23]. If the ER becomes stressed under growth conditions it might be useful to limit the transcriptional UPR initially, because a reduction in translation rates might largely suffice to restore homeostasis. Again, under these conditions low SKN-1 activity could act as a brake on the transcriptional UPR. With respect to the oxidative/xenobiotic stress response, it could be important for the ER to have a “vote” on its intensity, given the profound influence of SKN-1/Nrf on cellular redox status and resources devoted to the ER. It seems likely, therefore, that the ER not only manages its own homeostasis, but through SKN-1/Nrf has a broader impact on cellular stress defense networks that is likely to be critical in their normal and pathological functions.

## Materials and Methods

### Gene Expression Analysis

For each condition studied, RNA was extracted from approximately 100 µl of packed mixed-stage worms that were collected in M9 at the indicated time point. To induce UPR-associated gene expression, at day three of adulthood worms were treated with 5 µg/ml TM (Sigma) for 16 hours [15], or at day four with 5 mM DTT (Sigma) [54] for two hours, 5 µM thapsigargin (Enzo) [56] for two hours, or 5 µM Bortezomib (proteasome inhibitor, LC Labs) for six hours (similar to published *C. elegans* MG132 proteasome inhibitor treatment [57]). In each case, these treatments were non-lethal. For arsenite (AS) and tBOOH exposure, up to 100 µl of packed worms were collected and nutated in 5 mM AS or 12 mM tBOOH for 1 hour (a non-lethal duration). Each of these treatments was performed in a volume of 1 ml, and was followed by pelleting. RNA was analyzed by qRT-PCR as described, with values normalized to an internal standard curve for each amplicon [19], [44]. The same treatment conditions were used for ChIP experiments.

### Transgenic Reporter Scoring

Expression or nuclear accumulation of transgenic GFP proteins was scored as “low,” “medium,” or “high” essentially as published [19], or were quantified using ImageJ 1.45S.

### ChIP Lysates and Analysis

ChIP was performed essentially as described [19], [44]. 2 ml of packed mixed-stage worms were crosslinked with formaldehyde at room temperature for 20 minutes. After quenching, lysis, and determination of protein concentration, 1 mg/ml samples were frozen as aliquots at −80°C. The resolution of the assay was approximately 250–500 bp [44]. The monoclonal antibody FC4 [58] was used for SKN-1 ChIP experiments, as in previous ChIP analyses [19]. Other antibodies are described in the Supplemental Experimental Procedures. Analyses of intergenic regions and control genes (not shown) indicated that average signals of 14%, 11%, 26%, 4%, 11%, 7%, and 8% represent thresholds for specific presence of SKN-1, Pol II, PSer2, and H3-AcK56, XBP-1, ATF-6, and Histone H3 respectively.

### ER Fractionation

Worms from five confluent 20 cm^2^ plates were collected in M9 with or without TM treatment (5 µg/ml) for 16 hours, in order to generate 2× 1 ml of packed mixed-stage animals. Worms were sonicated 3× for 20 seconds in homogenization buffer (supplied by IMGENEX kit, supplemented with HDAC inhibitors, protease inhibitors, phosphatase inhibitors, and MG132) with the Branson midiprobe 4900 Sonifer before fractionation with the IMGENEX Endoplasmic Reticulum Enrichment Kit (Cat No. 10088K) [59]. Mitochondrial and ER fractions were washed 3× with 1 ml PBS and resuspended in 400 µl PBS (supplemented with HDAC, protease, and phosphatase inhibitors and MG132). Up to 100 µl of the ER or cytoplasmic fractions were used for each IP.

### Immunoprecipitation and Western Blotting

Controls for a polyclonal rabbit antiserum raised against SKN-1c (JDC7, referred to as pSKN-1) are shown in Figures S4F–S4J. HSP-3/4/BiP was detected with either C-terminal Drosophila Hsc3 [60] (Figures 6A and 6B) or N-terminal human BiP antibody (Sigma et21) [61], [62] (Figures 6C and 6E). Note that both BiP antibodies recognized the same 75 kD band. ATF-6 (Abcam ab11909), Tubulin (Sigma #9026), and GAPDH (Santa Cruz sc25778) antibodies were also used. Phosphorylated p38 was detected using an antibody from Cell Signaling T180/Y182 as described previously [23]. For Western blotting, antibodies were used at the following dilution: 1∶200 FC4 monoclonal αSKN-1, 1∶200 polyclonal αSKN-1, 1∶1000 αPol II, and 1∶1000 for αHsc3. All other antibodies were used at manufacturer's recommended concentrations.

For IPs, the indicated antibodies (50 µl FC4 monoclonal αSKN-1 or polyclonal αSKN-1,10 µl Hsc3 (BiP) or 20 µl BiP (Sigma)) and pre-blocked Salmon Sperm DNA/Protein A beads (Zymed) were added to lysates or samples from the fractionation described above. The final volume was brought to 500 µl in 1× PIC, 1× PMSF, and 1∶1000 MG132 diluted in 1× PBS. Samples were nutated overnight at 4°C and washed three times for 5 minutes at 4°C the next day with NP-40 wash buffer. Beads were spun down at 3000 rpm and resuspended in 4× SDS Laemmli Buffer. Samples were boiled for 15 minutes with 20 µl β-mercaptoethanol and 50 µl 4× SDS Laemmli. Samples were loaded (50 µl each) onto NuPAGE Novex Bis-Tris 10% Gels. Pierce ECL or Femto Western Blotting Substrate was used for detection.

Other methods are available in Text S1 (Supplementary Materials and Methods).

## Supporting Information

Figure S1 Distinct SKN-1 functions in ER and oxidative stress responses. (A) The unfolded protein response (reviewed by [5], [6]). Functions of the canonical UPR signaling and transcription factors, which are labeled according to *C. elegans* nomenclature, are discussed in the text. IRE-1 includes an endoribonuclease activity that initiates splicing of the *xbp-1* mRNA and degrades many ER-associated mRNAs, and a kinase domain that initiates signaling through stress-activated protein kinase (SAPK) pathways. The membrane kinase PERK (PEK-1 in *C. elegans*) inhibits translation by phosphorylating eIF2α. As a result, ATF4 (ATF-5) is translated preferentially. Cleavage of ATF-6 in the Golgi releases it into the cytoplasm, which allows it to accumulate in the nucleus. The ER chaperone BiP (HSP-3/4 in *C. elegans*) participates in regulating these canonical ER signaling proteins [10], [63]. In this study we show that during the UPR, SKN-1 is upregulated at the mRNA and protein levels, and binds to many of the same downstream target promoters as the other UPR transcription factors. Here we depict this by showing these factors all binding to the same promoter. These downstream targets include UPR signaling and transcription factor genes. SKN-1 is present in the cytoplasm and nucleus but also associates with the ER (see text). (B) Analysis of UPR markers over a time-course of TM treatment. The levels of *hsp-4* and unspliced and spliced *xbp-1* mRNAs (see Figures 3A and S1B) were assayed by qRT-PCR after treatment with tunicamycin (TM) at the non-lethal concentration of 5 µg/ml. As in a previous publication [15], 16 hours of 5 µg/ml TM treatment was selected for subsequent gene expression analyses that involved TM (both mRNA and ChIP). Shorter time courses were chosen for other ER stress treatments (see Materials and Methods). (C) TM-induced ER stress failed to activate many genes that are: 1) constitutively regulated by SKN-1 (K10B2.2, C35B1.5, F32A5.3, T06D8.8, Y40D12A.2, *gst-1*), and 2) upregulated by SKN-1 in response to oxidative stress (K10B2.2, C35B1.5, Y40D12A.2, and *gst-1*) [21]. W03A5.7 (*dnj-24*) is a control gene that is not induced by TM as previously reported [8]. qRT-PCR analyses of animals that were treated with Control pL4440 or *skn-1* RNAi are shown. Error bars represent SEM, * p-value≤.05, ** ≤.01, *** ≤.001 as calculated by student's t-test.
(TIF)

Figure S2 Direct regulation of downstream genes by SKN-1. (A–C) SKN-1 directly activates genes in response to arsenite (AS, oxidative) stress. Recruitment of endogenous SKN-1 to the site of transcription of *atf-5* (A), *pcp-2* (B), and *gst-4* (C) was assayed by ChIP. Error bars represent SEM; * p-value≤.05, ** ≤.01, *** ≤.001, calculated by student's t-test.
(TIF)

Figure S3 SKN-1-dependent activation of core UPR genes. (A) SKN-1 is required for ER stress (TM) to induce accumulation of total *xbp-1* mRNA. *skn-1* refers to *skn-1* RNAi, with analysis performed by qRT-PCR. (B, C) SKN-1 is required for TM-induced activation of *hsp-4p*::GFP. (B) Nomarski (top) and fluorescence (bottom) images show representative *hsp-4p*::GFP adults that had been exposed to either empty pL4440 vector (left) or *skn-1* RNAi (right), and treated with TM for 16 hrs. (C) SKN-1-dependence of TM-induced *hsp-4/BiP* promoter activation in the intestine. *hsp-4p*::GFP expression scoring is described in the Experimental Procedures (n≥100 worms), *** p-value<.0001 by chi^2^, (D, E) XBP-1 (D) and SKN-1 (E) bind to the *hsp-4/BiP* gene in response to ER stress, with binding detected by ChIP. Error bars represent SEM, * p-value≤.05, ** ≤.01, *** ≤.001 calculated by student's t-test for S3A, D-E.
(TIF)

Figure S4 Increased expression of SKN-1 and downstream genes in response to ER stress. (A) Increased expression of *skn-1* mRNAs in response to TM treatment. mRNAs that encode the indicated SKN-1 isoforms were assayed by qRT-PCR using appropriate specific primer sets. (B) Activation of SKN-1-regulated ER stress-associated genes by thapsigargin treatment (Thap, 5 µM, 2 hours), measured by qRT-PCR. (C) Upregulation of SKN-1 target genes in response to treatment with the proteasome inhibitor bortezomib (5 µM, 6 hours). (D–E) Upregulation of *skn-1* and selected *skn-1*-regulated genes in response to *hsp-4* (BiP) (D) or *atf-6* (E) downregulation, analyzed by qRT-PCR. (F) Increased expression of the SKN-1 protein in response to TM-induced ER stress. IP (immunoprecipitation)-Western analysis was performed with the FC4 α–SKN-1 monoclonal (mSKN-1), which was raised against bacterially-expressed SKN-1c and should detect all SKN-1 isoforms [19], [58]. The indicated SKN-1 band (arrow) corresponds to approximately the size predicted for the SKN-1a isoform (see text). Bands that correspond to IgG heavy chain (HC) and light chain (LC) are also indicated. (G) Reduction in SKN-1 levels in response to *skn-1* RNAi, assayed by IP-Western in which the IP was performed with a polyclonal SKN-1 antibody (pSKN-1, also raised against SKN-1c), and mSKN-1 was used for detection. Note that IP with an antibody against RNA Pol II did not non-specifically isolate this SKN-1 species (arrow). (H) Detection of bacterially-expressed GST-SKN-1c (arrow) by Western blotting with mSKN-1 (left) and pSKN-1 (right). (I) Detection of SKN-1 by Western blotting without IP. Lysates from TM-treated worms were analyzed. Note that both the mSKN-1 and pSKN-1 antibodies recognize the same 85 kD SKN-1 species (arrow), and that *skn-1* RNAi reduced its intensity. Tubulin is shown as a loading control. (J) Increased SKN-1 expression after DTT-induced ER stress. SKN-1 was immunoprecipitated using pSKN-1 (lanes 1, 2, and 4) or pre-immune serum (lane 3), then SKN-1 was detected by Western blotting with pSKN-1 (arrow). In lane 4, recombinant GST::SKN-1c that had been conjugated to sepharose beads was used to deplete the pSKN-1 antibody prior to IP (pre-clearing). Note that SKN-1 was not detected in the pre-immune IP or under pre-clear conditions. (K) DTT-induced activation of ER stress genes *hsp-4* and *atf-5* is *skn-1*-dependent. Control and DTT values are also shown in Figure 4C, but are compared here to expression in a *skn-1* RNAi sample that was generated in parallel. RNA was analyzed by qRT-PCR. (L) *skn-1* and *hsp-4* RNAi comparably decreased survival under conditions of reductive ER stress (treatment with 5 mM DTT). Because of sequence similarity, *hsp-4* RNAi may also affect *hsp-3*. See also Table S3. (M) Failure of TM to activate representative SKN-1 target genes that are upregulated by AS-induced oxidative stress, assayed by qRT-PCR as in (A–E). TM values are also shown in Figure S1C. (N) Failure of SKN-1 to accumulate in nuclei in response to ER stress. A representative experiment is shown in which SKN-1b/c::GFP levels were scored in intestinal nuclei after treatment with AS (n≥30), TM (n≥20), or DTT (n≥15) *** p-value<.0001 as determined by chi^2^ test. (O) Increased accumulation of oxidized proteins in *ire-1(RNAi)* animals. Oxidized proteins, an indirect indicator of ROS levels, were detected by Western blotting using the Oxyblot system (Millipore). A *C. elegans* lysate was treated with 2,4-dinitrophenylhydrazine (DNPH) to derivatize oxidized protein carbonyl sidechains to 2,4-dinitrophenylhydrazone (DNP-hydrazone), then analyzed by Western blotting with an antibody to DNP. Higher levels of oxidized proteins were associated with *ire-1* RNAi, but not with the non-lethal treatments with AS (5 mM) and TM (5 µg/ml) that were used for gene expression analyses. For Figures S4A–S4E, and S4K–S4M error bars represent SEM, * p-value≤.05, ** ≤.01, *** ≤.001 calculated by student's t-test.
(TIF)

Figure S5 Core UPR factors are required for SKN-1-mediated ER stress response. (A) Importance of XBP-1 for TM-induced SKN-1 target gene expression. RNAi was detected by qRT-PCR. (B–F) Canonical UPR factors are required for SKN-1-dependent gene activation that is induced by ER stress. (B, C) TM-induced SKN-1 binding and P-Ser2 Pol II accumulation at *atf-5* is abolished by *ire-1* RNAi. Recruitment of SKN-1 (D, F) and total Pol II (E, G) to *pcp-2* and *gst-4* was similarly impaired by *hsp-4* or *pek-1* RNAi. (H, I) Direct binding of endogenous XBP-1 at the *pcp-2* and *gst-4* loci, detected by ChIP. Note that TM treatment increased binding near the transcription start site. Possible elements that are characteristic of XBP-1 or ATF6/XBP1 binding [37] are present at the *skn-1*, *pcp-2*, and *gst-4* loci (not shown). (J, K) TM-induced recruitment of ATF-6 to *pcp-2* and *gst-4*, detected by ChIP. For A–K, error bars represent SEM, * p-value≤.05, ** ≤.01, *** ≤.001 calculated by student's t-test.
(TIF)

Figure S6 Evidence for association of SKN-1 with the ER. (A) A predicted transmembrane domain (a.a. 39–59) is present near the N-terminus of SKN-1a (Phobius algorithm). This transmembrane domain was also predicted by six additional transmembrane algorithms: 1) DAS, 2) MEMstat, 3) HMMTOP, 4) Mobyl, 5) TMAP, and 6) TMHMM (from SDSC Workbench). (B) Isolation of an ER-enriched fraction from *C. elegans*. Total cytoplasmic lysate, ER, and crude Mitochondrial fractions were Western blotted for HSP-3/4 and GAPDH (cytoplasm marker). Note the absence of GAPDH and the enrichment of HSP-3/4 relative to GAPDH in the ER fraction, and the exclusion of HSP-3/4 from the mitochondrial fraction in Figure 6C.
(TIF)

Figure S7 Importance of UPR signaling for SKN-1-mediated responses to oxidative stress. (A) *xbp*-1 is required for AS to induce expression of *skn-1* and downstream SKN-1 targets. Expression was assayed by qRT-PCR under Control or *xbp-1* RNAi conditions. (B) AS-induced upregulation of *gcs-1p::GFP* was impaired in *ire-1(RNAi)* animals. For each set, n≥57. (C) Many genes that were induced by SKN-1 in response to TM (Figures 1A and 1B, Table S1) were not upregulated by AS. (D) AS-induced accumulation of SKN-1::GFP in nuclei requires *ire-1*. IRE-1 expression was inhibited by RNAi. For each set, n≥39. (E, F) Survival of the indicated stress treatments was impaired by knockdown of core UPR factor genes. See also Table S4. For (A), (C), (E), and (F), error bars represent SEM and * p-value≤.05, ** ≤.01, *** ≤.001 calculated by student's t-test, # not significant. For S7B and S7D, *** p-value<.0001 by chi^2^.
(TIF)

Table S1 SKN-1 appears to regulate many genes that are involved in ER- or UPR-related functions (ER stress, ER maintenance, oxidative stress, and redox homeostasis). Genes were determined or predicted to be regulated by SKN-1 by microarray expression profiling [21], or genome-scale ChIP of transgenically-expressed SKN-1 [34]. The indicated genes have all been implicated in ER- or UPR-related functions [8], [52], [55], [64]–[88] and/or were found to be upregulated during the UPR [8], [15], [51], [66], [70], [89], [90].
(PDF)

Table S2 Individual Tunicamycin (TM) stress survival trials, shown as a composite in Figure 3H. Assay numbers represent parallel experiments. The *skn-1(zu67)* allele was used in each experiment, and in each case control was the wild type. All treatments were performed with adult worms, treated with either DMSO vehicle (-) or 35 µg/ml TM for seven days and then scored for survival by prodding with a pick. Survival is depicted as the percentage of animals that were alive at a given time point. Percent survival change refers to the difference between the control and *skn-1* survival percentages. Statistics are described in Figure 3H.
(PDF)

Table S3 Individual DTT stress survival trials, shown as a composite in Figure S4F. In each experiment, the indicated genes were knocked down by RNAi that was initiated at Day 1 of adulthood, with *pL4440* empty vector used as the control. Day 4 adult worms were treated with 5 mM DTT for 24 hours, then scored for survival. Number of treatment animals in parentheses refer to initial worm count before experiment. Survival percentages and differences are indicated as in Table S2. Statistics are described in Figure S4F.
(PDF)

Table S4 Individual Oxidative Stress survival trials, depicted as composites in Figures 7E, S7E, and S7F. Assay numbers represent parallel experiments. For assays 7 and 8, RNAi was initiated at the L1 stage, then Arsenite treatment was administered on Day 4 of adulthood. Survival was scored 36 hours later. Statistics are described in Figure 7. For assays 9 and 10, RNAi treatment was performed at Day 1 of adulthood, then Paraquat or tBOOH treatment was initiated at Day 4 of adulthood. Statistics are described in supplemental Figures S7E and S7F.
(PDF)

Text S1 Supplementary Materials and Methods.
(PDF)
