# Supplementary material for: Integration of the Unfolded Protein and Oxidative Stress Responses through SKN-1/Nrf
Source: PLoS Genet. 2013 Sep 12;9(9):e1003701. doi: 10.1371/journal.pgen.1003701 (PMC3772064; doi:10.1371/journal.pgen.1003701)
Supplement: Table S1 — SKN-1 appears to regulate many genes that are involved in ER- or UPR-related functions (ER stress, ER maintenance, oxidative stress, and redox homeostasis). Genes were determined or predicted to be regulated by SKN-1 by microarray expression profiling [21], or genome-scale ChIP of transgenically-expressed SKN-1 [34]. The indicated genes have all been implicated in ER- or UPR-related functions [8], [52], [55], [64]–[88] and/or were found to be upregulated during the UPR [8], [15], [51], [66], [70], [89], [90]. (PDF) [file pgen.1003701.s008.pdf]

Table S1.

**ER Stress and ER homeostasis**		
**Gene ID**	**Function**	**SKN-1 target study**
abu-8	alternative unfolded protein response [51]	this study
abu-11	alternative unfolded protein response [51]	this study
acl-4	Phospholipid biosynthesis gene, ER stress upregulated [8]	ChIP [34]
arf-1.1	GTP-binding ADP-ribosylation factor, ER stress upregulated [51]	ChIP [34]
atf-5	ER and oxidative stress transcription factor ATF4 [8,15]	ChIP [34], microarray [21]
atf-6	ER transcription factor [8]	ChIP [34]
B0348.2	LPS-induced tumor necrosis factor alpha factor, integral to membrane [8]	microarray [21]
C47E12.3	putative EDEM factor (ERAD) [8]	ChIP (operon) [34]
cdc-48.2	ERAD ATPase, p97 [8]	ChIP [34]
cdr-4	membrane bound gst, ER stress upregulated [8,51]	ChIP [34]
cebp-2	C48E7.11, putative C/EBPβ, ER stress regulated [81]	ChIP [34]
cept-1	PtdCho synthesis, ER membrane integrity [80,88]	ChIP [34]
cka-1	PtdCho synthesis, ER membrane integrity [80,88]	ChIP [34]
ckb-4	PtdCho synthesis, up with ER stress [8] and oxidative [21], ER membrane integrity [80,88]	ChIP [34], microarray [21]
cnb-1	calcineurin, UPR [64], interacts with PERK [65], not predicted to increase with ER stress [8]	ChIP [34]
crt-1	calreticulin, matainence of ER [66], up with ER stress (this study)	ChIP [34]
dnj-28	ER chaperone, regulated with ER stress [8]	ChIP [34]
E04D5.1	putative eIF2α (Wormbase), PERK phosphorylation target during ER stress [89]	ChIP [34]
erd-2	putative ER lumen protein retaining receptor similar to yeast *erd2* [67]	ChIP [34], microarray [21]
ero-1	ER oxidoreductase, upregulated with ER stress [8,15]	ChIP [34]
F41B4.3	Membrane bound, ER stress upregulated [51]	ChIP [34]
F42G8.8	putative serine/threonine specific PP1 [68] (by homology, Wormbase)	microarray [21]
F47A4.5	Phospholipase, ER stress upregulated [8]	ChIP [34]

F55E10.6	putatitve corticosteroid beta-dehydrogenase (by homology, Wormbase), most likely resides in ER [82]	microarray [21]
fzo-1	Mito/ER connector (Mfn1/Mfn2) essential for mitochondrial-associated membrane (MAM) formation [70]	ChIP [34]
hsp-16.11	chaperone, ER stress upregulated [51]	ChIP [34]
hsp-16.2	chaperone, ER stress upregulated [51]	ChIP [34]
hsp-16.41	chaperone, ER stress upregulated [51]	ChIP [34]
hsp-3	ER chaperone, BiP homolog, upregulated with ER stress [8,51]	ChIP [34]
hsp-4	ER chaperone, BiP homolog, upregulated with ER stress [8,51,90]	this study
ire-1	ER signaling factor [90]	ChIP [34]
larp-1	homologous to La protein, stabilizes yeast Hac1(*xbp-1*) transcript [71]	ChIP [34]
larp-5	homologous to La protein, stabilizes yeast Hac1 (*xbp-1*) transcript [71]	ChIP [34]
lgg-1	homologous to LC3/Atg8, regulated by ER stress factor ATF4 [72]	ChIP [34]
lgg-3	homologous to ATG12, regulated by ATF4 during ER stress [73]	ChIP [34]
lin-15B	negatively regulates G1 progression, ER stress upregulated [51]	ChIP [34]
lpd-2	regulates *lpd* genes, modulates ER stress [74,83]	ChIP (operon) [34]
memb-2	Sec22 SNARE protein, ER stress upregulated [8]	ChIP [34]
pcp-2	Prolyl Carboxy Peptidase like, IRE-1 dependent [8], up with oxidative stress	microarray [21]
pcyt-1	CCT enzyme for PtdCho synthesis, ER membrane integrity [80,88]	ChIP [34]
pdi-2	ER Protein disulfide isomerase, upregulated with ER stress [8,51]	ChIP [34]
pdi-3	ER protein disulfide isomerase [75]	ChIP [34]
pek-1	ER signaling factor, kinase [8]	ChIP [34]
psd-1	flow of phospholipid mito to ER, MAM associated [76]	ChIP [34]
R05G6.7	VDAC, MAM associated [52], not predicted to increase with ER stress [8]	ChIP [34]
R12E2.13	Mannosyltransferase, upregulated with ER stress [8]	ChIP [34]
sca-1	SERCA, calcium homeostasis, upregulated with ER stress [8]	ChIP [34]
sec-22	Sec22 SNARE protein, ER stress upregulated [8]	ChIP [34]
sel-1	HMG-CoA Reductase, ERAD complex, upregulated with ER stress [8]	ChIP [34]

skn-1	ER stress factor, up with ER stress (this study, [8])	ChIP [34]
T05E11.3	HSP90 chaperone [66], up with ER stress (this study)	ChIP [34]
T14G8.3	HSP70-like ER resident, ER stress upregulated [51]	ChIP [34]
tag-320	cell redox homeostasis, thioredoxin, ER stress upregulated [51]	ChIP [34]
vha-13	vacuolar proton-translocating ATPase (V-ATPase), regulated by calcineurin/calreticulin [77]	ChIP [34], microarray [21]
xbp-1	ER transcription factor, up with ER stress (this study, [90])	ChIP [34]
Y40B10A.2	O-methyltransferase, ER stress upregulated [51]	ChIP [34]
Y41C4A.11	Similar to COPI, WD repeat family, ER stress upregulated [8,51]	ChIP [34], microarray [21]
Y51B9A.9	putative MAPK9 like kinase (JNK pathway, by homology, Wormbase) [84]	microarray [21]

Endobiotic and Xenobiotic metabolism, predicted to be ER-localized

**Gene ID**	**Function**	**SKN-1 target study**
C35D10.10	putative TMX thioredoxin-like protein, ER Golgi transport, MAM (by homology, Wormbase) [78]	microarray [21]
C46H11.2	Flavin-containing monooxygenase, most likely ER localized [55]	microarray [21]
cyp-13A1	cytochrome p450, most likely ER localized [55]	ChIP [34]
cyp-13B1	cytochrome p450, most likely ER localized [55]	microarray [21]
cyp-14A1	cytochrome p450, most likely ER localized [55]	microarray [21]
cyp-25A4	cytochrome p450, most likely ER localized [55]	ChIP [34]
cyp-33C4	cytochrome p450, most likely ER localized [55]	ChIP [34]
cyp-33C7	cytochrome p450, most likely ER localized [55]	ChIP [34]
cyp-33C8	cytochrome p450, most likely ER localized [55]	ChIP [34]
cyp-43A1	cytochrome p450, most likely ER localized [55]	ChIP [34]
F17A9.4	potential NADH oxidase, predicted SR localized [85,86]	ChIP [34], microarray [21]
F17A9.5	potential NADH oxidase, predicted SR localized [85,86]	microarray [21]
F56D5.3	potential NADH oxidase, predicted SR localized [85,86]	microarray [21]
hpo-19	NADH-cytochrome b-5 reductase, integral to membrane, predicted ER localized [87]	ChIP (operon) [34], microarray [21]
K04A8.10	UDP-Glucuronosyl Transferase, ER lumen [79]	microarray [21]

ugt-1	UDP-Glucuronosyl Transferase, ER lumen [79]	microarray [21]
ugt-11	UDP-Glucuronosyl Transferase, ER lumen [79]	ChIP [34]
ugt-13	UDP-Glucuronosyl Transferase, ER lumen [79]	ChIP [34]
ugt-16	UDP-Glucuronosyl Transferase, ER lumen [79]	microarray [21]
ugt-20	UDP-Glucuronosyl Transferase, ER lumen [79]	ChIP [34]
ugt-22	UDP-Glucuronosyl Transferase, ER lumen [79]	ChIP [34], microarray [21]
ugt-23	UDP-Glucuronosyl Transferase, ER lumen [79]	microarray [21]
ugt-31	UDP-Glucuronosyl Transferase, ER lumen [79]	ChIP [34], microarray [21]
ugt-46	UDP-Glucuronosyl Transferase, ER lumen [79]	microarray [21]
ugt-48	UDP-Glucuronosyl Transferase, ER lumen [79]	microarray [21]
ugt-58	UDP-Glucuronosyl Transferase, ER lumen [79]	ChIP [34], microarray [21]
ugt-64	UDP-Glucuronosyl Transferase, ER lumen [79]	ChIP [34]
ZK742.4	potential NADH oxidase, predicted SR localized [85,86]	ChIP [34], microarray [21]
