# Supplementary material for: Integration of the Unfolded Protein and Oxidative Stress Responses through SKN-1/Nrf
Source: PLoS Genet. 2013 Sep 12;9(9):e1003701. doi: 10.1371/journal.pgen.1003701 (PMC3772064; doi:10.1371/journal.pgen.1003701)
Supplement: Table S2 — Individual Tunicamycin (TM) stress survival trials, shown as a composite in Figure 3H. Assay numbers represent parallel experiments. The skn-1(zu67) allele was used in each experiment, and in each case control was the wild type. All treatments were performed with adult worms, treated with either DMSO vehicle (-) or 35 µg/ml TM for seven days and then scored for survival by prodding with a pick. Survival is depicted as the percentage of animals that were alive at a given time point. Percent survival change refers to the difference between the control and skn-1 survival percentages. Statistics are described in Figure 3H. (PDF) [file pgen.1003701.s009.pdf]

Table S2.

Strain	Treatment	Treatment Survival (%)	No. Treatment animals	Control Survival (%)	No of Control Animals	% Survival Change	Assay #
skn-1	-	88.5	52	97.1	103	-8.6	1
skn-1	TM	57.4	61	97.1	103	-39.7	1
skn-1	-	100	31	100	78	0	2
skn-1	TM	70.7	41	97.5	79	-26.8	2
skn-1	-	85.7	35	92.7	41	-7.0	3
skn-1	TM	21.4	14	74.2	31	-52.8	3
skn-1	-	97.4	39	100	75	-2.6	4
skn-1	TM	51.2	43	93.8	65	-42.6	4
skn-1	-	100	50	100	48	0.0	4
skn-1	TM	49.4	79	97.4	38	-48	4
