# Supplementary material for: Integration of the Unfolded Protein and Oxidative Stress Responses through SKN-1/Nrf
Source: PLoS Genet. 2013 Sep 12;9(9):e1003701. doi: 10.1371/journal.pgen.1003701 (PMC3772064; doi:10.1371/journal.pgen.1003701)
Supplement: Table S3 — Individual DTT stress survival trials, shown as a composite in Figure S4F. In each experiment, the indicated genes were knocked down by RNAi that was initiated at Day 1 of adulthood, with pL4440 empty vector used as the control. Day 4 adult worms were treated with 5 mM DTT for 24 hours, then scored for survival. Number of treatment animals in parentheses refer to initial worm count before experiment. Survival percentages and differences are indicated as in Table S2. Statistics are described in Figure S4F. (PDF) [file pgen.1003701.s010.pdf]

Table S3.

RNAi	Treatment	Treatment Survival (%)	No. Treatment animals	Control Survival (%)	No of Control Animals	% Survival Change	Assay #
skn-1	DTT	60.6	33	76.9	52	-16.3	5
hsp-4	DTT	42.9	7(30)	76.9	52	-34.0	5
skn-1	DTT	47.7	86	74.1	81	-26.4	6
hsp-4	DTT	68.8	16(20)	74.1	81	-5.3	6
