# Supplementary material for: Integration of the Unfolded Protein and Oxidative Stress Responses through SKN-1/Nrf
Source: PLoS Genet. 2013 Sep 12;9(9):e1003701. doi: 10.1371/journal.pgen.1003701 (PMC3772064; doi:10.1371/journal.pgen.1003701)
Supplement: Table S4 — Individual Oxidative Stress survival trials, depicted as composites in Figures 7E, S7E, and S7F. Assay numbers represent parallel experiments. For assays 7 and 8, RNAi was initiated at the L1 stage, then Arsenite treatment was administered on Day 4 of adulthood. Survival was scored 36 hours later. Statistics are described in Figure 7. For assays 9 and 10, RNAi treatment was performed at Day 1 of adulthood, then Paraquat or tBOOH treatment was initiated at Day 4 of adulthood. Statistics are described in supplemental Figures S7E and S7F. (PDF) [file pgen.1003701.s011.pdf]

Table S4.

Strain	RNAi	Treatment Timepoint	Treatment Survival (%)	No. Treatment animals	Control Survival (%)	No of Control Animals	% Survival Change	Assay #
SJ30 ire-1 (zc14)	*hsp-4*	AS, 5mM, 36 hr	0	20	82.5	75	-82.5	7
SJ30 ire-1 (zc14)	*hsp-4*	AS, 5mM, 36 hr	0	37	82.5	75	-82.5	7
SJ17 xbp-1 (zc12)	*ire-1*	AS, 5mM, 36 hr	0	49	82.5	75	-82.5	7
SJ17 xbp-1 (zc12)	*ire-1*	AS, 5mM, 36 hr	0	51	82.5	75	-82.5	7
SJ30 *ire-1 (zc14)*	*xbp-1*	AS, 5mM, 36 hr	61.3	53	82.5	75	-21.2	7
SJ30 *ire-1 (zc14)*	*xbp-1*	AS, 5mM, 36 hr	67.8	47	82.5	75	-14.7	7
SJ30 ire-1 (zc14)	*hsp-4*	AS, 5mM, 36 hr	0	36	69.5	59	-69.5	8
SJ30 ire-1 (zc14)	*hsp-4*	AS, 5mM, 36 hr	0	36	69.5	59	-69.5	8
SJ30 ire-1 (zc14)	*xbp-1*	AS, 5mM, 36 hr	0	19	69.5	59	-69.5	8
SJ30 ire-1 (zc14)	*xbp-1*	AS, 5mM, 36 hr	0	16	69.5	59	-69.5	8
SJ17 *xbp-1 (zc12)*	*ire-1*	AS, 5mM, 36 hr	0	17	69.5	59	-69.5	8
SJ17 *xbp-1 (zc12)*	*ire-1*	AS, 5mM, 36 hr	0	18	69.5	59	-69.5	8

VC1099 *hsp-4 (gk514)*	*hsp-4*	AS, 5mM, 36 hr	0	22	69.5	59	-69.5	8
VC1099 *hsp-4 (gk514)*	*hsp-4*	AS, 5mM, 36 hr	0	27	69.5	59	-69.5	8
	hsp-4	Para, 20mM, 10 days	14.5	55	33.8	343	-19.3	9
	hsp-4	Para, 20mM, 10 days	7	100	33.8	343	-26.8	9
	hsp-4	Para, 20mM, 10 days	18.5	54	33.8	343	-15.3	9
	xbp-1	Para, 20mM, 10 days	14.3	28	33.8	343	-19.5	9
	xbp-1	Para, 20mM, 10 days	13.9	36	33.8	343	-19.9	9
	xbp-1	Para, 20mM, 10 days	25	32	33.8	343	-8.8	9
	xbp-1	tBOOH, 10mM, 10 hr	55.6	9	83.7	59	-21.2	10
	xbp-1	tBOOH, 10mM, 10 hr	66.7	30	83.7	59	-12.3	10
	hsp-4	tBOOH, 10mM, 10 hr	33.3	21	83.7	59	-33.7	10
	hsp-4	tBOOH, 10mM, 10 hr	48.1	27	83.7	59	-21.8	10
