# Supplementary material for: Integration of the Unfolded Protein and Oxidative Stress Responses through SKN-1/Nrf
Source: PLoS Genet. 2013 Sep 12;9(9):e1003701. doi: 10.1371/journal.pgen.1003701 (PMC3772064; doi:10.1371/journal.pgen.1003701)
Supplement: Text S1 — Supplementary Materials and Methods. (PDF) [file pgen.1003701.s012.pdf]

TEXT S1.

C. elegans and strains

C. elegans were maintained at 20°C on NGM plates on the streptomycin-resistant *E. coli* strain OP50-1 (Caenorhabditis Genetics Center (CGC)), unless otherwise indicated. The wild-type strain was N2 Bristol. The deletion mutants *atf-5(tm4397)*, *hsp-4(gk514)*, and *pek-1(ok275)*, and the missense mutations *xbp-1(zc12)* and *ire-1(zc14)* were obtained from the CGC, along with the *hsp-4p::GFP* promoter-fusion reporter strain [15]. The transcriptional SKN-1 target gene reporter *gcs-1::GFP* and the *SKN-1b/c::GFP* translational fusion were described previously [33]. *SKN-1b/c:GFP* encodes two of the three SKN-1 isoforms (SKN-1b and SKN-1c).

RNAi

Animals were synchronized with bleach and transferred onto HT115 bacteria expressing dsRNA on day 1 of adulthood, and analyzed after 3 days of feeding. *pL4440* was used as the control. RNAi clones were obtained from the *C. elegans* Orfeome library [91].

RNA extraction and qRT-PCR

TRI Reagent (Sigma-Aldrich) was added to all samples to a total volume of 300 µl. RNA was extracted after 5 flash freeze cycles at -80°C, then treated with DNAse1 (Roche). For qRT-PCR, cDNA was synthesized using the Superscript III kit (Lot No. 1002006, Life Technologies), and SYBR GreenER signal (Life Technologies) was

detected and quantified using Applied Biosystems 7900HT Sequence Detection System. mRNA fold inductions were calculated relative to control genes (*mtce-23*, *gpd-2, his-10,* or *tba-1*) that were determined not to be induced by these treatments (based upon normalization to worm count).

ChIP antibodies

In addition to SKN-1 (see main Materials and Methods), IPs were performed with antibodies against phosphorylated CTD Ser2 (PSer2), total Pol II CTD, Histone H3 [44], mouse XBP-1 (sc7160) [37,90], human ATF-6 (ab11909) [92], and acetylated Histone H3 (Abcam ab76307) [93]. The last antibody most likely detects H3K56ac, H3K4ac, H3K9ac, and H3K27ac primarily [94].

GFP reporter analysis

Animals were treated with TM, AS, or DTT essentially as described for mRNA extraction. After suspension in Levamisole, images were captured using a Zeiss Axioskop 2 microscope and AxioVision 4.8.2 Software. For DTT, AS, and TM treatments, GFP intensity was quantified using ImageJ, or by eye as high, medium and low essentially as described. [17,33,47]. For *gcs-1::GFP* and *hsp-4::GFP*, ‘‘High’’ indicated GFP detection at high levels throughout most of the intestine, while ‘‘Medium’’ referred to animals with robust GFP signal present only anteriorly or posteriorly. For SKN-1b/c::GFP, “High” indicated that a strong SKN-1::GFP signal was present in all intestinal nuclei, and “Medium” that nuclear SKN-1::GFP was present at high levels anteriorly, posteriorly or both, but barely visible midway through the intestine, or that a weak signal was observed in all intestinal nuclei.

Stress Assays

All stress assays were performed at 20ºC. In TM experiments, approximately 20-30 worms were plated in triplicate, and treated with 35 µg/ml tunicamycin at day 1 of adulthood by layering 500 µl of TM (dissolved in DMSO) diluted in M9. For AS and tBOOH survival, ≥ 9 day 4 adult worms per RNAi condition were picked into 96 well plates containing 5 mM AS or 10 mM tBOOH in M9, and were scored at the indicated time points. For DTT survival ≥ 20 worms were incubated in 1 ml 5 mM DTT diluted in M9, nutating for 24 hours, followed by pelleting and plating on empty plates for scoring. 20 mM paraquat treatment was performed on plates mixed with indicated RNAi food for 10 days.

SUPPLEMENTARY REFERENCES

91. Rual JF, Ceron J, Koreth J, Hao T, Nicot AS, et al. (2004) Toward improving *Caenorhabditis elegans* phenome mapping with an ORFeome-based RNAi library. Genome Res 14: 2162-2168.
92. Ou YC, Yang CR, Cheng CL, Raung SL, Hung YY, et al. (2007) Indomethacin induces apoptosis in 786-O renal cell carcinoma cells by activating mitogen-activated protein kinases and AKT. Eur J Pharmacol 563: 49-60.
93. Lo KA, Bauchmann MK, Baumann AP, Donahue CJ, Thiede MA, et al. (2011) Genome-wide profiling of H3K56 acetylation and transcription factor binding sites in human adipocytes. PLoS One 6: e19778.
94. Drogaris P, Villeneuve V, Pomies C, Lee EH, Bourdeau V, et al. (2012) Histone deacetylase inhibitors globally enhance H3/H4 tail acetylation without affecting H3 lysine 56 acetylation. Sci Rep 2: 220.
